# A missense mutation converts the Na^+^,K^+^-ATPase into an ion channel and causes therapy-resistant epilepsy

**DOI:** 10.1016/j.jbc.2021.101355

**Published:** 2021-10-28

**Authors:** Sofia Ygberg, Evgeny E. Akkuratov, Rebecca J. Howard, Fulya Taylan, Daniel C. Jans, Dhani R. Mahato, Adriana Katz, Paula F. Kinoshita, Benjamin Portal, Inger Nennesmo, Maria Lindskog, Steven J.D. Karlish, Magnus Andersson, Anna Lindstrand, Hjalmar Brismar, Anita Aperia

**Affiliations:** 1 Neuropediatric Unit, Department of Women’s and Children's Health, Karolinska Institutet, Stockholm, Sweden; 2 Centre for Inherited Metabolic Diseases (CMMS), Karolinska University Hospital, Stockholm, Sweden; 3 Department of Applied Physics, Science for Life Laboratory, Royal Institute of Technology, Stockholm, Sweden; 4 Department of Biochemistry and Biophysics, Science for Life Laboratory, Stockholm University, Stockholm, Sweden; 5 Department of Molecular Medicine and Surgery, Center for Molecular Medicine, Karolinska Institutet, Stockholm, Sweden; 6 Department of Neurobiology, Care Sciences and Society, Karolinska Institutet, Stockholm, Sweden; 7 Department of Chemistry, Umeå University, Umeå, Sweden; 8 Department of Biomolecular Sciences, Weizmann Institute of Science, Rehovoth, Israel; 9 Department of Pharmacology, Institute of Biomedical Science, University of São Paulo, São Paulo, Brazil; 10 Department of Pathology, Karolinska University Hospital, Stockholm, Sweden; 11 Department of Clinical Genetics, Karolinska University Hospital, Stockholm, Sweden; 12 Department of Women’s and Children's Health, Science for Life Laboratory, Karolinska Institutet, Stockholm, Sweden

**Keywords:** Na,K-ATPase, arginine mutation, epilepsy, *de novo* mutation, leak channel, CADD, combined annotation-dependent depletion, DOPC, dioleoylphosphatidylcholine, ExAC, Exome Aggregation Consortium, GFP, green fluorescent protein, MAP2, microtubule-associated protein 2, MRI, magnetic resonance imaging, OS, ouabain-sensitive, OR, ouabain-resistant, SNV, single nucleotide variant, WGS, whole-genome sequencing

## Abstract

The ion pump Na^+^,K^+^-ATPase is a critical determinant of neuronal excitability; however, its role in the etiology of diseases of the central nervous system (CNS) is largely unknown. We describe here the molecular phenotype of a Trp931Arg mutation of the Na^+^,K^+^-ATPase catalytic α1 subunit in an infant diagnosed with therapy-resistant lethal epilepsy. In addition to the pathological CNS phenotype, we also detected renal wasting of Mg^2+^. We found that membrane expression of the mutant α1 protein was low, and ion pumping activity was lost. Arginine insertion into membrane proteins can generate water-filled pores in the plasma membrane, and our molecular dynamic (MD) simulations of the principle states of Na^+^,K^+^-ATPase transport demonstrated massive water inflow into mutant α1 and destabilization of the ion-binding sites. MD simulations also indicated that a water pathway was created between the mutant arginine residue and the cytoplasm, and analysis of oocytes expressing mutant α1 detected a nonspecific cation current. Finally, neurons expressing mutant α1 were observed to be depolarized compared with neurons expressing wild-type protein, compatible with a lowered threshold for epileptic seizures. The results imply that Na^+^,K^+^-ATPase should be considered a neuronal locus minoris resistentia in diseases associated with epilepsy and with loss of plasma membrane integrity.

Epileptic encephalopathies are severe brain disorders that generally arise in infancy and cause developmental delay and sometimes early death. Seizures are often resistant to treatment and lead to cognitive decline. The etiology of epileptic encephalopathies is multifactorial, ranging from acquired structural deficits, such as stroke to congenital or genetic causes that may result in altered membrane potential, failure to propagate neuronal signals correctly, death of single neurons, and/or loss of neuronal networks (1, 2). In recent years, the availability of exome and genome sequencing has assisted the identification of epilepsy of genetic origin and highlighted the role of *de novo* dominant disease-causing variants in sporadic epileptic encephalopathies. The majority of mutated genes are directly involved in regulation of neuronal activity. Such genes include *SCN1A* and *SCN8A*, which encode voltage-gated sodium channels that initiate the action potential, and *KCNQ2* and *KCNT1*, which encode voltage-gated potassium channels and contribute to restoration of the resting membrane potential after neuronal activity (3, 4, 5). Voltage-gated sodium and potassium channels are the major determinants of neuronal electricity, together with the ion pump Na,K-ATPase (6). By transporting three Na^+^ ions out of the neuron and two K^+^ ions into the neuron at the expense of one ATP molecule, Na,K-ATPase builds and maintains the Na^+^ and K^+^ electrochemical gradients that are central for the membrane potential.

Na,K-ATPase-mediated ion transport accounts for approximately 50% of total brain energy consumption (7). Yet only a few studies have investigated the electrogenic role of Na,K-ATPase in neurological diseases (5). Mutations of *ATP1A1,* encoding the ubiquitous catalytic subunit α1, and *ATP1A3,* encoding the neuron specific catalytic subunit α3 (8), are rare and associated with epilepsy in some, but not all, cases (9). Mutations of *ATP1A3* are often associated with alternating hemiplegia in childhood (10, 11), a severe neurological disease with onset in childhood, and rapid-onset dystonia parkinsonism, a movement disorder with onset in adulthood (12). Mutation of *ATP1A1* has been identified in adrenal adenomas of patients presenting with hyperaldosteronism and hypertension due to increased intracellular sodium concentration (13, 14). *ATP1A1*-associated neurological diseases have been reported in 42 individuals from seven families with symptoms compatible with Charcot–Marie–Tooth syndrome (15) and three nonrelated children with a mutation associated with epilepsy of varying severity and hypomagnesemia (16). The difference in the clinical presentations reported in patients with *ATP1A1* mutations indicates that both the position and nature of the substituted amino acid may be responsible for epileptic activity and that studies of the molecular phenotype of the mutated α1 subunit can provide information about the role of Na,K-ATPase electrogenicity in diseases of the central nervous system.

Here, we describe the atomic phenotype of a *de novo* missense variant of *ATP1A1* in an infant with recurrent therapy-resistant epileptic seizures who died at 10 months of age. The mutation, Trp931Arg (W931R), was located in the eighth transmembrane helix. Functional characterization of the mutation revealed an abnormal inward current, similar to that observed in the two previously reported cases of *ATP1A1* mutations associated with epilepsy. Since an arginine residue located in the plasma membrane can attract water molecules (17), we performed molecular dynamics simulations to examine whether the W931R mutation compromised the integrity of ion-binding sites in Na,K-ATPase *via* water accumulation. Our study underscores the importance of describing the molecular and atomic phenotypes of mutations in genetic epilepsy and sets the stage for new strategies to develop therapeutic tools for these devastating conditions.

## Results

### Clinical history

**Figure 1:**
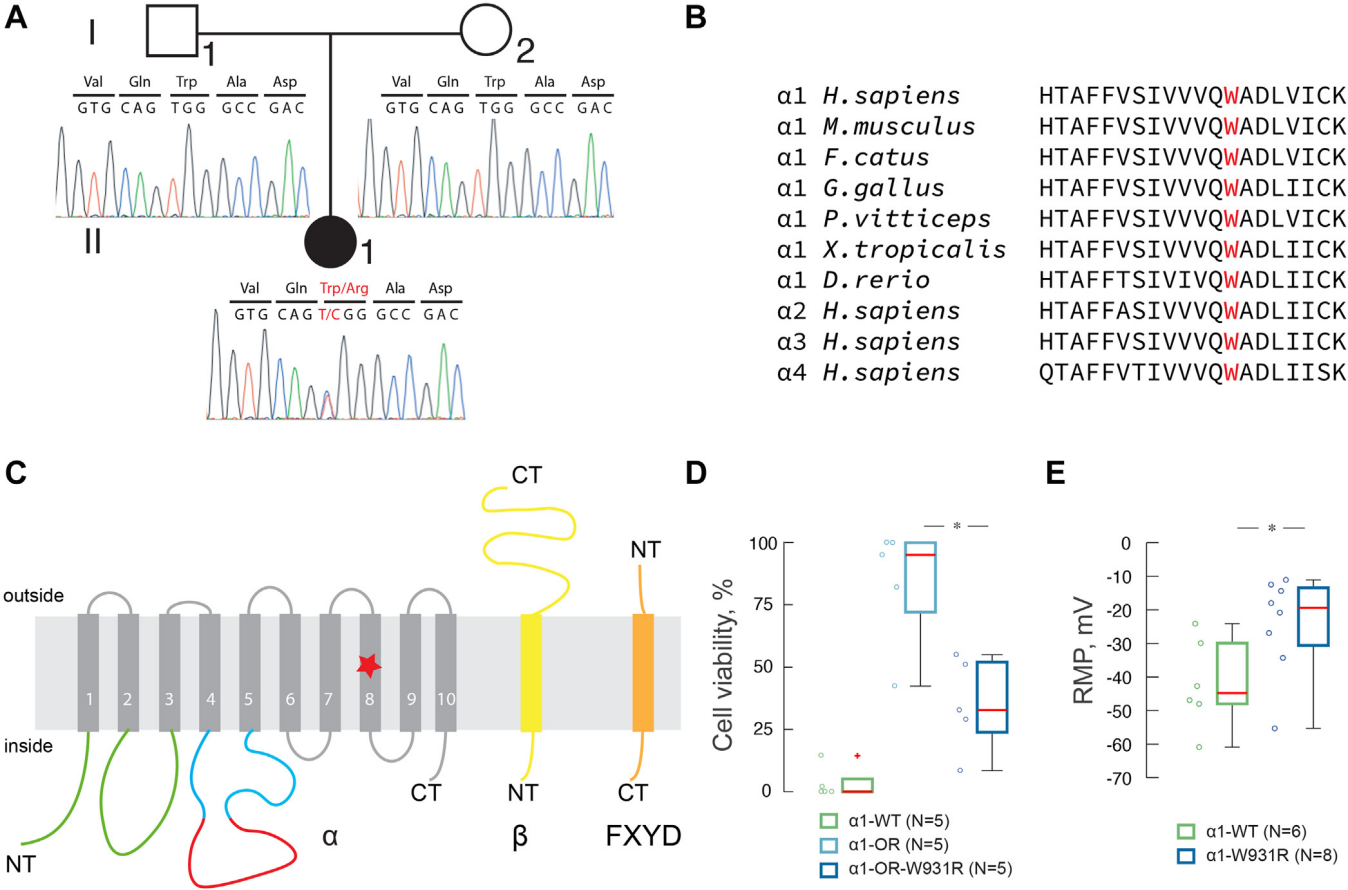
**Variant of *ATP1A1* in a patient with fatal epilepsy.***A*, pedigree of the affected patient II-1 and her parents I-1 and I-2. Sanger sequencing chromatograms show the *de novo* variant c.2791T > C (W931R) in the *ATP1A1* gene of the affected patient II-1. *B*, multiple alignment of the protein sequence of the eighth transmembrane domain of the Na,K-ATPase α subunit from different species. The residue W931 (*red*) is highly conserved between α1 subunits of vertebrates and between other human α subunits (α2, α3, and α4). *C*, schematic representation of the α (*gray*), β (*yellow*), and FXYD (*orange*) subunits. The α subunit domains: A- (*green*), N- (*cyan*), and P- (*red*). The W931R variant is located in the eighth transmembrane domain of the α1 subunit. *D*, the viability of HEK293a cells expressing the W931R α1 mutant is significantly lower than that of HEK293a cells expressing WT α1. HEK293a cells were transfected with α1-WT (WT α1 subunit, ouabain-sensitive), α1-OR (α1 subunit, ouabain-resistant), or α1-OR-W931R (α1 subunit, ouabain-resistant, with the W931R variant) and treated with ouabain (10 μM). Viability is expressed as number of cells in ouabain treated condition normalized to the untreated condition. *E*, the resting membrane potential (RMP) in rat hippocampal neurons transfected with α1-WT or α1-W931R. The resting membrane potential is significantly higher in α1-W931R-transfected cells than in α1-WT-transfected cells. Mann–Whitney test, ∗*p* < 0.01.

The affected infant was the first child of healthy, nonconsanguineous parents (Fig. 1*A*). The pregnancy was uneventful. She was born at term with a normal birthweight and did not present any abnormal morphological features. At 3 weeks of age, choreatic movements of the tongue and hands were noted. At 3 months of age, she suffered her first general tonic-clonic seizure, a status epilepticus that required intensive care. The seizure was preceded by a period of poor feeding and weight loss. Magnetic resonance imaging (MRI) of the brain was normal and a subsequent electroencephalogram revealed no seizure activity. Her development up to this point was age-appropriate. She suffered her next status epilepticus at 4 months of age and another at 4.5 months of age, both of which required intensive care. From 5 months of age, she suffered numerous refractory status epilepticus and remained hospitalized until her death at 10 months of age. Among the multiple antiepileptic drugs that were tested, only ketamine elicited a small, but transient, effect. After each seizure, she lost physical and cognitive abilities, which were initially regained during seizure-free intervals. Electroencephalograms showed multifocal seizure activity after 4 months of age, and MRI showed signs of brain atrophy with enlarged ventricles at 6 months of age. She had no cardiac symptoms, and her heart was structurally and functionally normal at 5 months of age. Contrast-enhanced MRI showed that the thorax and abdomen were normal. Extensive metabolic investigations, including muscle biopsy, did not reveal any pathology (Text S1). She had profound hypomagnesemia (0.42–0.71 mM serum concentration) due to high urinary magnesium excretion. Correction of her blood magnesium level did not affect the frequency or duration of seizures. Autopsy did not show any macroscopic pathology; however, microscopic examination of brain tissue revealed swelling and loss of some hippocampal cells and Purkinje cells in the cerebellum.

### Identification of the W931R mutation

Using whole-genome sequencing (WGS), we identified a *de novo* mutation (NM_000701.7:c.2791T>C; p.W931R) in exon 20 of *ATP1A1* (Fig. 1*A*). *ATP1A1* encodes the α1 subunit of Na,K-ATPase, which comprises ten transmembrane helices (TM1–10). Na,K-ATPase consists of a catalytic α-subunit, a regulatory β-subunit and is also often associated with a regulatory FXYD protein (Fig. 1*C*). The α-subunit has ten transmembrane domains (TM1-TM10) with N- and C-terminus located in the cytoplasm. The transmembrane domains are α-helixes and form binding sites for transported ions. In mammals, there are four isoforms of the catalytic α-subunit (α1–α4) each of which has different affinities to sodium, potassium, and ATP.

The highly conserved W931 residue is located in TM8 (Fig. 1, *B* and *C*). The W931R mutation is predicted to be highly deleterious with a Combined Annotation-Dependent Depletion (CADD GRCh37-v1.6) c-score of 29.9 (18). In addition, an Exome Aggregation Consortium (ExAC) pLI score of 1.0 (19) suggests that this gene is highly intolerant to loss-of-function mutations, and a z-score of 6.90 for missense variants indicates that this gene has increased resistance to variation. No additional variants in other genes encoding Na^+^ or K^+^ channels were identified. The variants identified by WGS were first filtered for a minor allele frequency of less than 0.01% in ExAC (19) and SweGen (20). Variants with low quality and located in repetitive regions were filtered out. Next, we filtered for different inheritance models in the trio WGS data, keeping only those that were *de novo*, homozygous, and compound heterozygous. The pathogenicity of each variant was then evaluated using CADD (18), PolyPhen2 (21), SIFT (22), and MCAP (23). Finally, the molecular and biological function of each gene as well as its association with a genetic disease was evaluated. After thorough filtering and evaluation, only eight variants remained and *ATP1A1* was the most plausible candidate gene in this patient (Table S1).

To assess the pathogenicity of the W931R mutation, we performed an ouabain survival assay in which the capacities of wild-type (WT) and mutant α1 subunits to support cell survival were compared (12). Cells that expressed mutant α1 had a significantly lower viability than cells that expressed WT α1 (Fig. 1*D*).

### Immunofluorescence imaging of brain autopsy sections

**Figure 2:**
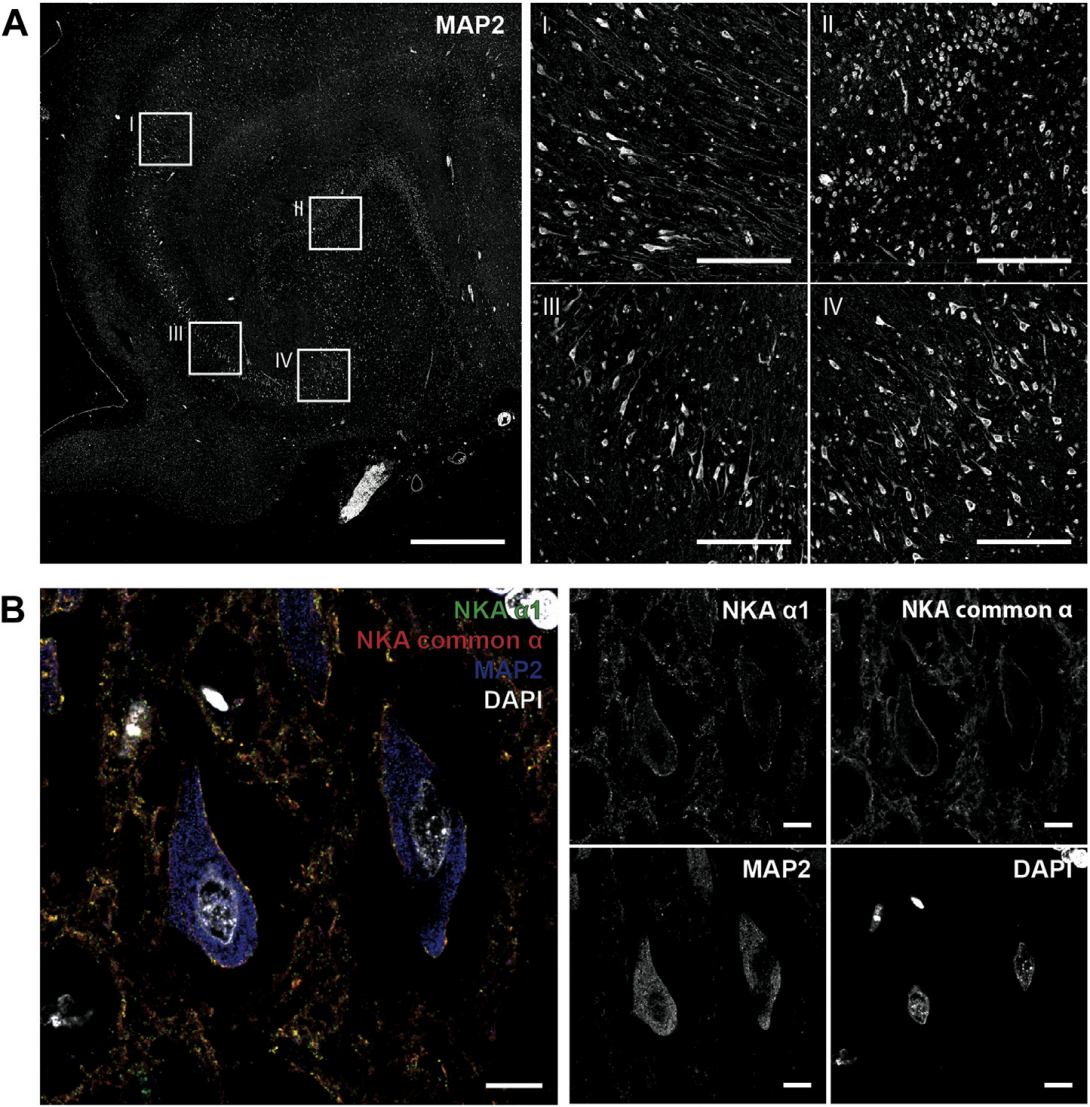
**Confocal microscopy of the affected hippocampus.***A*, confocal microscopy of the hippocampus stained for the neuronal marker MAP2 shows the overall distribution of neurons in the hippocampus (overview, *left*) and the specific morphologies of different types of neurons (close-ups I–IV, *right*). Scale bars: 1 mm (overview, *left*) and 100 μm (close-ups, *right*). *B*, confocal microscopy of individual hippocampal neurons stained for the Na,K-ATPase (NKA) α1 subunit, the Na,K-ATPase common α subunit, MAP2, and nuclei (DAPI) reveals the membrane localization of the common α subunit and particularly the α1 subunit. Scale bars: 10 μm.

To investigate if the α1 subunit was expressed in neurons of the patient’s brain and if the mutation caused a major loss of neurons, we carried out immunofluorescence imaging of labeled tissue sections of the hippocampus using confocal microscopy (Fig. 2). Residual neurons were detected in hippocampal sections (Fig. 2*A*). To visualize expression of the α1 subunit, we focused on individual neurons in the hippocampus (Fig. 2*B*). Staining for the α1 subunit demonstrated that it localized to the membrane in mature neuronal (microtubule-associated protein 2 (MAP2)-positive) cells. However, this could be attributable to expression of the WT allele due to the heterozygous expression of the W931R variant. Comparison of the distributions of the α1 subunit and total α subunits showed that a subset of the total α subunit content colocalized with the α1 subunit, indicating the presence of other α subunits.

### Effect of the W931R mutation on the resting membrane potential

The effect of the W931R mutation on the neuronal resting membrane potential was studied by performing single-cell patch-clamp recordings of rat hippocampal neurons expressing WT or mutant human α1 fused to green fluorescent protein (GFP) to identify transfected neurons. The resting membrane potential was −19.71 ± 3.17 mV in neurons expressing mutant α1, which was less negative than that in neurons expressing WT α1 (−42.09 ± 5.43 mV; *p* = 0.008) (Fig. 1*E*).

### Low plasma membrane expression of W931R α1

**Figure 3:**
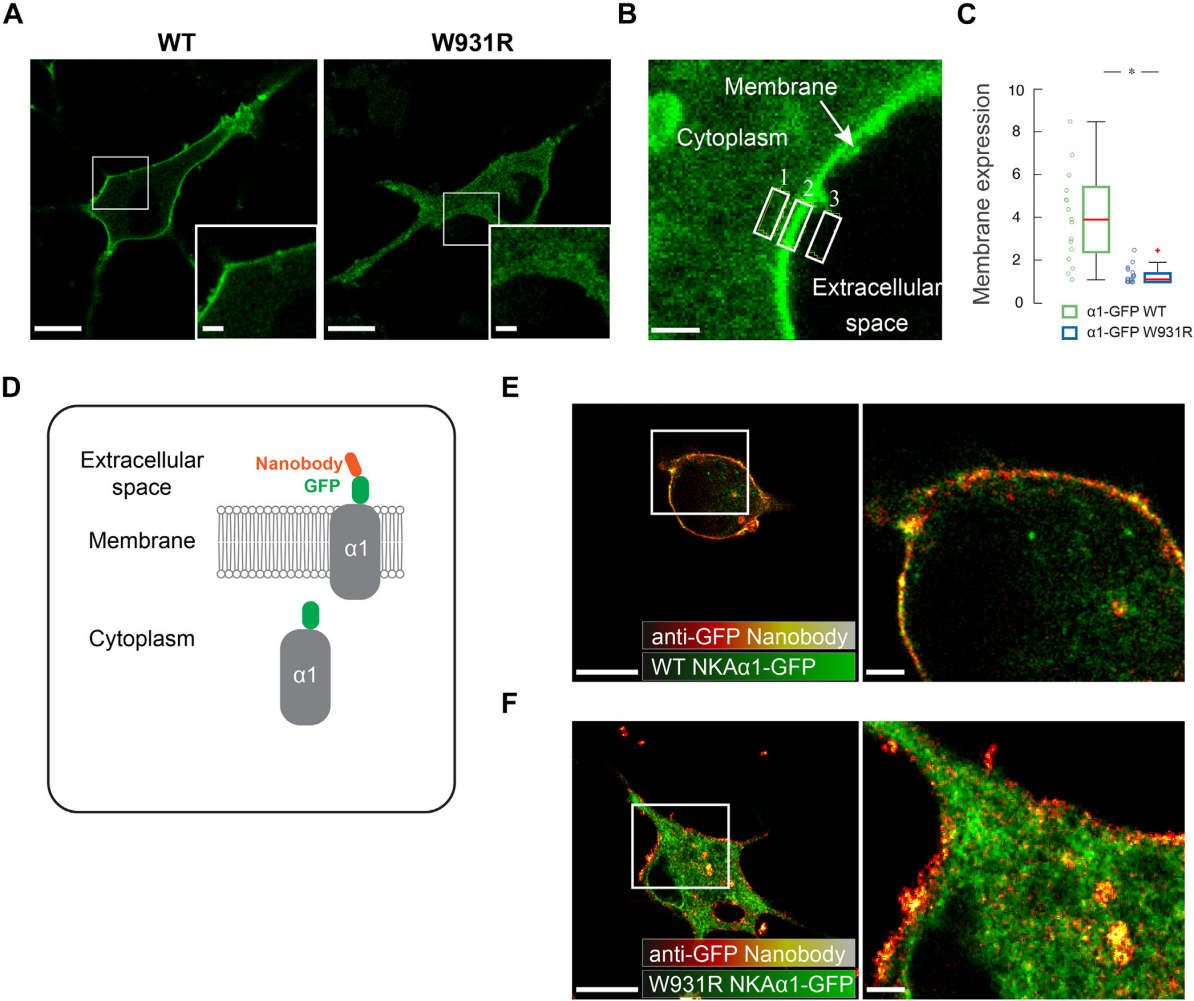
**The W931R variant affects the membrane localization of Na,K-ATPase.***A*, rat hippocampal neurons expressing WT and W931R α1 genetically tagged with extracellular GFP. Representative images show that GFP localizes to the membrane in cells expressing WT α1, but this localization is less distinct in cells expressing W931R α1. Scale bars: 10 μm, magnified 2 μm. *B*, membrane expression of the respective protein was calculated as the ratio of the average fluorescence intensity in the plasma membrane (2) to the average fluorescence intensity in the cytoplasm (1) corrected for background signals (3). Scale bar: 1 μm. *C*, at 2 days after transfection of neurons, the membrane localization of W931R α1 is impaired compared with that of WT α1 (two-sided Wilcoxon rank sum test, ∗*p* < 0.001). *D*, schematic representation of cells expressing a GFP-tagged protein labeled with an anti-GFP nanobody. Only proteins inserted into the plasma membrane can be labeled with the anti-GFP nanobody because it is added to live cells. *E*, membrane labeling with an anti-GFP nanobody (*fire*) shows distinct labeling of WT α1 and colocalization with GFP in the membrane. Scale bars: 10 μm, magnified 2 μm. *F*, only a fraction of the nanobody and GFP colocalize at the plasma membrane in cells expressing W931R α1. *Green* indicates GFP. Scale bars: 10 μm, magnified 2 μm.

To examine the trafficking of the W931R α1 variant to the plasma membrane, we carried out transient expression of WT or mutated α1 fused to GFP in cultured rat hippocampal neurons. WT α1 exhibited a clear membrane localization, whereas the W931R α1 variant was localized to both the cytoplasm and plasma membrane (Fig. 3*A*). Quantification revealed that plasma membrane expression of W931R α1 variant was significantly lower than that of WT α1 (Fig. 3, *B* and *C*). This finding was consistent at 1, 2, and 3 days after transfection, showing that plasma membrane expression of W931R α1 variant was not simply delayed (Fig. S1). This effect did not appear to be neuron-specific since it was also observed in other cell types (Fig. S2).

GFP was fused in the third extracellular loop to determine insertion of the WT and mutated proteins into the plasma membrane by performing live-cell immunofluorescence imaging of extracellular GFP. An anti-GFP nanobody was used to detect extracellularly exposed GFP and so to identify α1 inserted into the plasma membrane (Fig. 3, *D* and *F*). In hippocampal neurons transfected with WT α1-GFP, signals of GFP and the anti-GFP nanobody were detected in the plasma membrane (Fig. 3*E*). In cells transfected with W931R α1-GFP, distribution of the GFP signal was diffuse and was detected in both cytoplasm and plasma membrane. The anti-GFP nanobody signal was only detected in the plasma membrane, indicating that a fraction of the W931R α1 variant was located within the plasma membrane.

### Nonselective cation leak currents in oocytes expressing W931R α1

**Figure 4:**
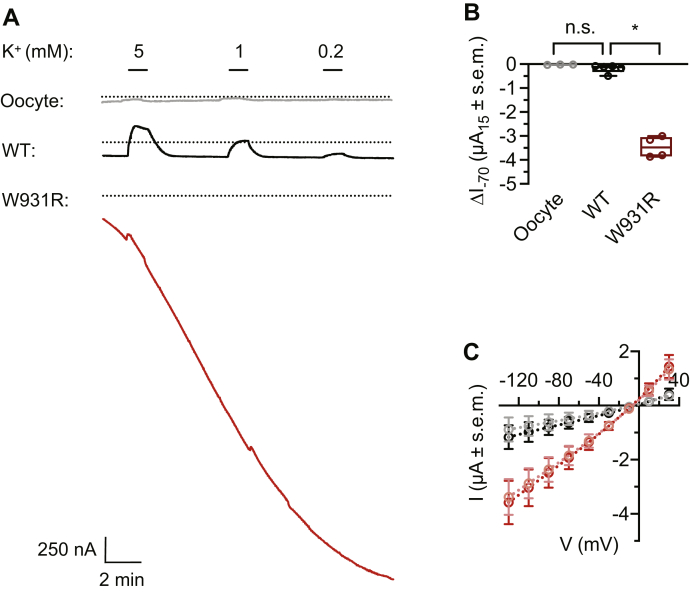
**Electrophysiology in W931R α1-expressing cells.***A*, representative traces from two-electrode voltage-clamp electrophysiology recordings at −70 mV in *Xenopus* oocytes overexpressing human Na,K-ATPase, showing concentration-dependent activation of outward currents upon exposure to external K^+^ with minimal baseline drift over 15 min in oocytes injected with WT α1 mRNA. By contrast, oocytes injected with W931R α1 mRNA exhibit an escalating inward current. *B*, mean baseline displacement after clamping for 15 min at −70 mV, following the protocol in (*A*). Columns represent absolute currents (μA), n = 5; significance relative to WT α1, two-tailed unpaired *t* test, ∗*p* < 0.01. *C*, current–voltage relationships under resting conditions (100 mM Na^+^, 0 mM K^+^) for noninjected control oocytes (*black*) and W931R α1-injected oocytes (*red*), n ≥ 3. Treatment with 10 μM ouabain is represented by a lighter shade of *black* and *red* for noninjected and W931R α1-injected oocytes, respectively.

**Figure 5:**
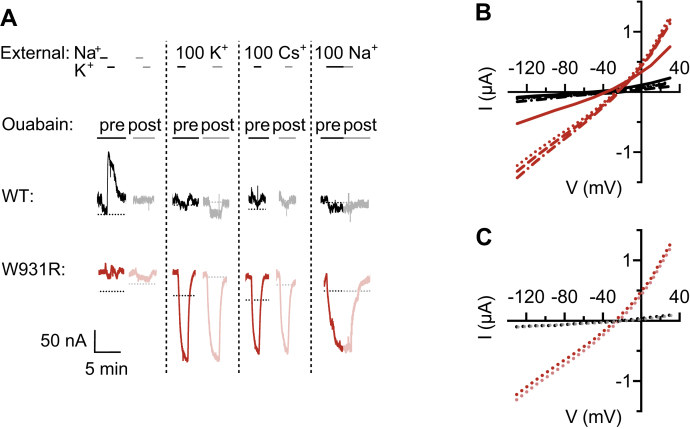
**Nonselective pump-deficient leak currents in W931R α1-expressing cells.***A*, sample traces at −30 mV show ouabain-sensitive K^+^-activated outward currents from WT pumps, and ouabain-insensitive leak currents in the presence of various cations from mutant (W931R) pumps. Na,K-ATPase activity was activated in NMDG^+^ media by perfusing cells with 10 mM Na^+^ for 1 min immediately followed by 10 mM K^+^ for 1 min; other ions (Na^+^, K^+^, and Cs^+^) were introduced at a concentration of 100 mM for the indicated durations. Individual traces are baseline-adjusted for clarity; *dashed horizontal* guides indicate a current of 0 nA. *B*, sample current–voltage relationships from the experiment in (*A*), showing comparable mutant leak currents at all voltages for Na^+^ (*dot*), K^+^ (*dash-dot*), and Cs^+^ (*dash*) relative to NMDG^+^ buffer (*solid line*). *C*, sample current–voltage relationships from the experiment in (*A*), showing no change in Na^+^ leak currents before or after 10 μM ouabain treatment. In (*A*–*C*), *black* and *red* represent WT and mutant α1, respectively. Treatment with 10 μM ouabain is represented by a lighter shade of *black* or *red*.

To characterize the electrophysiological properties of the W931R variant, we expressed WT and mutant α1 in *Xenopus laevis* oocytes for up to 1 week and performed voltage-clamp current measurements at −70 mV. In oocytes expressing WT α1, we recorded a concentration-dependent outward current in response to 5 mM external K^+^ (304 ± 29 nA; Fig. 4*A*). The endogenous Na,K-ATPase current in response to 5 mM external K^+^ was measured in noninjected oocytes (control) and was significantly weaker (19 ± 3 nA; Fig. 4*A*). In oocytes expressing mutated α1, the current measurements were dominated by an escalating inward current, which resulted in microampere-scale baseline currents after voltage-clamp for ≥15 min (Fig. 4, *A* and *B*). During the escalating current, a small response was observed when external K^+^ was applied. The baseline-corrected amplitude of the response to K^+^ was of the same magnitude as the response of endogenous Na,K-ATPase in control cells (Fig. 4*A*). To measure leak currents, we reduced the voltage-clamp potential to −25 mV and used a pulse protocol. Leak currents were consistently larger in oocytes expressing mutated α1 than in control oocytes (Fig. 4*C*). Treatment with ouabain at a concentration that blocks the pump function of Na,K-ATPase (10 μM) did not affect the leak currents. To further characterize the leak currents, we exposed oocytes to pulses of 100 mM Na^+^, K^+^, or Cs^+^ in NMDG^+^ media. In oocytes expressing mutated α1, all ions induced inward currents with comparable amplitudes, indicating that any of these ions could mediate the leak (Fig. 5, *A* and *B*). The leak currents were not affected by treatment with 10 μM ouabain (Fig. 5, *A* and *C*). The electrophysiological recordings indicate that the W931R variant facilitates a nonselective cation leak under a range of conditions and disrupts the Na,K-ATPase pump activity. Loss of function has previously been described in Na,K-ATPase α1 mutations identified in adrenal adenomas (24).

### W931R α1 is inactive

For comparison with the expression and functional effects observed in animal cells and oocytes, we analyzed the expression and activity of the W931R variant in the yeast *P.pastoris.* This yeast species has previously been used for biochemical analyses of Na,K-ATPase isoforms, FXYD proteins, and mutants (25, 26, 27).

We measured the Na,K-ATPase activity (Fig. S3*B*) and found that the mutant protein has no detectable enzyme activity and essentially no specific ouabain binding, consistent with the observation in oocytes.

The maximal expression of the mutant at 20 °C was 20 ± 2.9% compared with expression of wild-type at 24 °C. In addition, for the mutant protein we observed fragments with lower mass than for the α subunit (Fig. S3*A*). These observations indicate that the mutant is unfolded, unstable, and susceptible to cellular degradation at 20 °C.

### A water pathway between the W931R mutation and the cytoplasm

**Figure 6:**
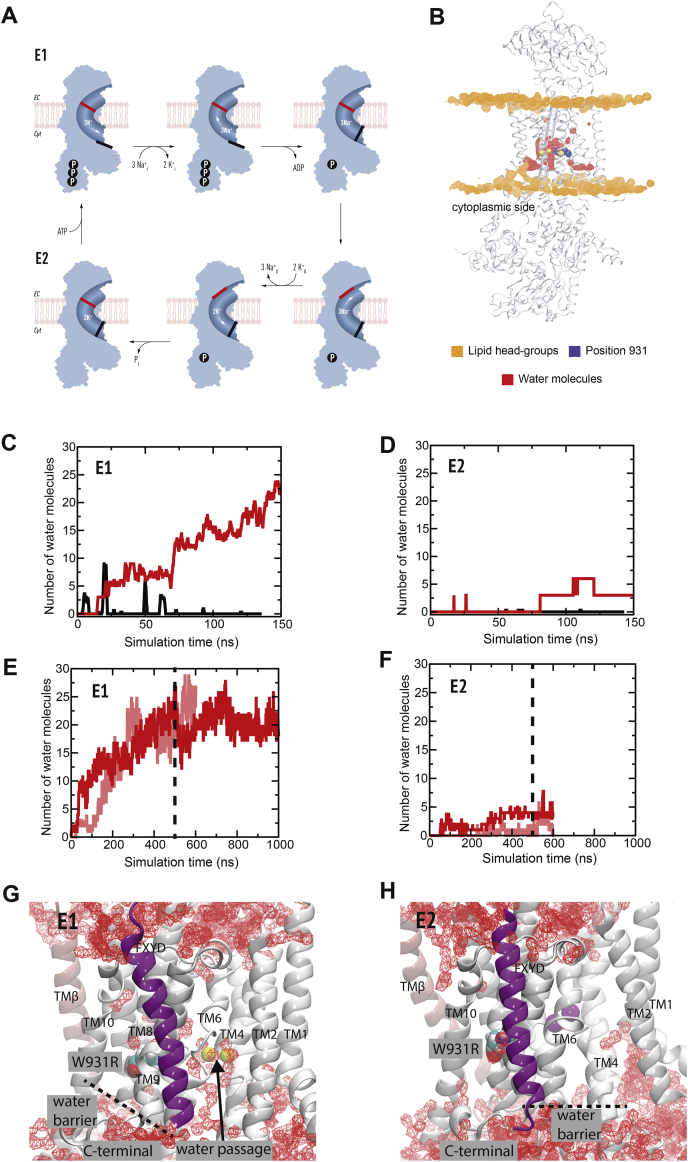
**Molecular dynamics simulations reveal water accumulation in the transmembrane domain.***A*, the Post-Albers scheme of the Na,K-ATPase transport cycle: In the E1 Na^+^ transporting state ATP and Na^+^ enter the protein from the cytoplasm and Na^+^ will be occluded (*black gate*). The protein is phosphorylated and Na^+^ is then released to the extracellular site. The protein is transformed into the E2 state that binds and occludes extracellular K^+^ (*red gate*). K^+^ is released into the cytoplasm, and the protein is transformed into the E1 Na^+^ and ATP binding state again. *B*, the average simulated structure of W931R Na,K-ATPase in the E1 state (*white*) with iso-density surfaces of lipid phosphates (*orange*) and water (*red*) at occupancies of 34% and 11%, respectively. Na^+^ ions (*yellow*) and the mutated arginine residue (*blue*) are shown as vdW *spheres*. *C* and *D*, the number of water molecules in the WT (*black*) and W931R mutant (*red*) simulations within 5 Å of position 931 in the (*C*) E1 and (*D*) E2 states. *E* and *F*, the number of waters within 3 Å of the (*E*) Na^+^-binding and (*F*) K^+^-binding residues for two repeat simulations (*red* and *light red*). *G* and *H*, water iso-density surfaces from the final 100 ns of the simulation depicted at 5% occupancy (*red*) corresponding to water within a 22 Å × 22 Å × 42 Å box spanning the membrane section and centered at the center-of-mass of the binding ions for the (*G*) E1 and (*H*) E2 state trajectories in the plasma membrane mimic. The location of the Arg931 variant is shown in licorice, and the sodium and potassium ions are colored *yellow* and *brown*, respectively. The FXYD protein is shown in *magenta*.

The catalytic α subunit of Na,K-ATPase undergoes large-scale conformational changes as it switches between outward transport of three Na^+^ ions in the E1 state to inward transport of two K^+^ ions in the E2 state (Fig. 6*A*). Arginine insertion into biological membranes can introduce water pores and destabilization (17). To explore whether arginine-mediated water accumulation could be the cause of the leak currents, we performed molecular dynamics simulations of WT and W931R variant proteins in the E1 (PDB ID: 4HQJ) and E2 (PDB ID: 3KDP) states in a neat dioleoylphosphatidylcholine (DOPC) lipid bilayers. While water did not accumulate in the WT protein, an abundance of water molecules was observed in the mutated protein (Fig. 6*B*). Water accumulated throughout the E1 state simulation (Fig. 6*C*), but not in the E2 state simulation (Fig. 6*D*).

To examine the effect of hydration on the ion-coordinating sites, we performed two parallel independent simulations for the mutated protein in the E1 and E2 states. Multicomponent symmetric lipid bilayers were used to mimic the plasma membrane environment, and a potential of 100 mV was applied after equilibration for 500 ns to reproduce a shift in the membrane potential*.* Water molecules entered the transmembrane domain throughout the E1 state simulation. Water accumulated close to the arginine mutation (Fig. 6*G*) and within 3 Å of the Na^+^-binding sites (Figs. 6*E* and S4*C*), but did not accumulate close to the K^+^-binding sites (Fig. 6, *F* and *H*).

**Figure 7:**
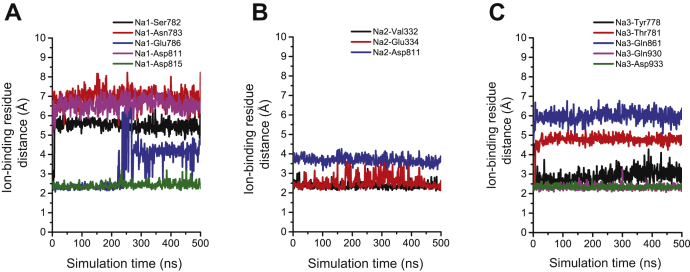
**Binding site dynamics and Na**^**+**^**ion stability.** Distances between the transported ions and the ion-coordinating residues in (*A*) site I (Na1), (*B*) site II (Na2), and (*C*) site III (Na3) in the E1 state simulation of W931R.

Several ion–amino acid interactions observed in the crystal structure were disrupted, and the Na^+^-binding residue Glu786 was displaced to directly interact with water after 200 ns in the E1 state simulation (Fig. 7*A*). In one simulation, a Na^+^ ion was lost from the binding site through a water pore and released into the cytoplasm after the 100 mV potential was applied (Fig. S5). In the E2 state stimulation, the ion-binding sites did not directly contact water molecules (Fig. S4*D*), and K^+^ ions were maintained within their binding positions (Fig. S4, *A* and *B*). The Arg931 residue faced away from the K^+^-binding sites and was packed closer to the surrounding lipids (Figs. 6*H* and S4*D*). None of the structures underwent large-scale conformational changes throughout the simulation (Fig. S6).

**Figure 8:**
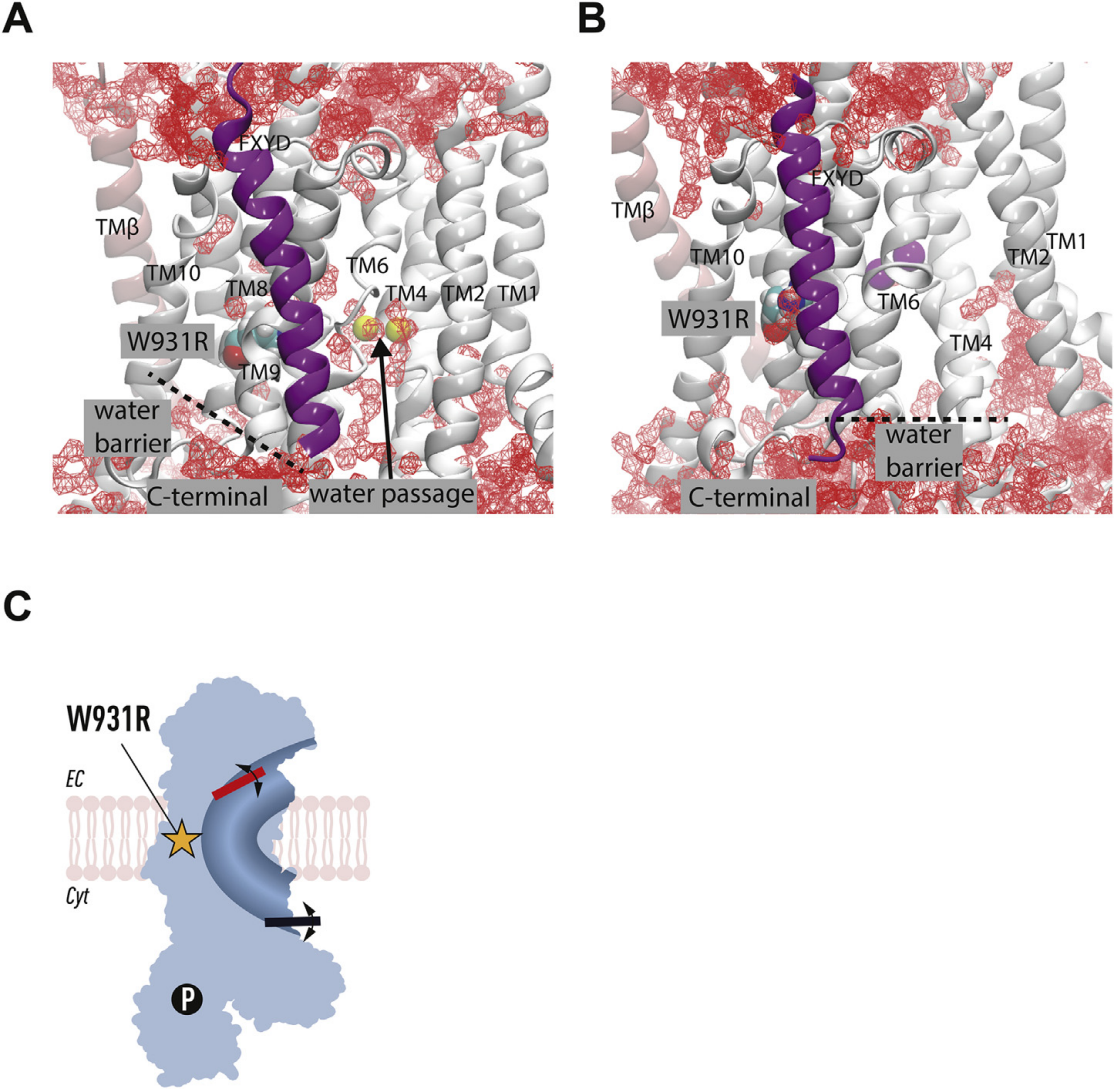
**Hydration of Na,K-ATPase leads to a switch in function from pump to unspecific cation channel.** The molecular dynamics simulations show a water pathway between TM helices 4 and 6 in the (*A*) E1 state from the cytoplasm to the arginine variant. Isosurface for 10% occupancy of the water oxygens is shown in *red*. The (*B*) E2 state is devoid of water along the corresponding pathway. *C*, proposed model for how the two gate lock in the Na,K-ATPase is corrupted by the W931R variant, the gates are disconnected, and the function of the protein is effectively converted into an unspecific cation channel.

Furthermore, the water molecules around the ion coordinating sites were supplied *via* a water pathway formed between the cytoplasm and the arginine mutation in the E1 state (Fig. 8*A*). Such a water pathway will likely allow ion leakage and thus provides a structural explanation for the transition of the Na,K-ATPase pump into a nonspecific cation channel. Surprisingly, the simulations did not show any water molecules entering *via* the C-terminus of the α subunit, which has a high affinity for sodium and has been suggested to be an intracellular sodium entry site (28, 29).

## Discussion

We describe here the case of an infant with severe therapy-resistant epilepsy and progressive encephalopathy who was diagnosed with a W931R mutation in the Na,K-ATPase catalytic α1 subunit. Na,K-ATPase α1 is expressed in all cells. This infant had few symptoms in organs other than the brain, except for a reduced capacity of the kidneys to retain magnesium. In neurons the membrane potential is determined by the electrochemical gradients of ions across the plasma membrane that is mainly mediated by voltage regulated Na^+^ and K^+^ channels and the Na,K-ATPase. The membrane potential sets a threshold for neuronal activity and action potentials and makes neurons particularly vulnerable to changes in membrane potential that can evoke epileptic activity (6, 30).

Molecular dynamics simulations of the principal states during Na,K-ATPase transport demonstrated that water molecules surrounded the mutation and ion-coordinating sites. Electrophysiological recordings in oocytes expressing mutated α1 demonstrated nonspecific ion leak currents. Based on these findings, we attribute the epileptic seizures to loss of the Na,K-ATPase-specific two-gate transport system and conversion of this pump into a nonspecific ion channel, resulting in leak currents that compromise the capacity to restore electrochemical gradients and the control of neuronal membrane potential.

Na,K-ATPase transports three Na^+^ ions out of the cell and two K^+^ ions into the cell *via* three ion-coordinating sites that can be accessed from only one side of the membrane at a time (Fig. 6*A*). The exact molecular localizations of the ion-coordinating sites change during the transition between the E1 and E2 states. The amino acids that coordinate Na^+^ and K^+^ transport are to a large extent the same for sites 1 and 2, which are accessed by both Na^+^ and K^+^ (29, 31). Site 3, on the other hand, is Na^+^-specific. The ion-transporting pathways are strictly controlled by coupled gates (32) that alternatively open and close to transport Na^+^ in the E1 state and K^+^ in the E2 state. This gating system is driven by energy released from ATP hydrolysis and distinguishes active transporters, such as the Na,K-ATPase ion pump, from ion channels, which generally require only one gate (33, 34). Our molecular dynamics simulations demonstrated that water molecules surrounded the ion-coordinating sites, particularly site 1. Hydration around the ion-coordinating sites has previously been reported in MD simulations of α1 mutations in adrenal adenomas (35). The enhanced hydration and the disturbance of ion coordination with an occasional ion loss indicate that the gating capacity in the mutant Na,K-ATPase is compromised. This will lead to loss of the pump-specific alternating transport of Na^+^ and K^+^ and leak of cations *via* the water pathway from the ion-coordinating sites to the cytoplasm.

Several water pathways have been observed to penetrate the membrane domain of the wild-type protein in MD simulations. In addition to an extracellular pathway (32, 36, 37), N-terminal (31, 37), C-terminal pathways (37, 38, 39), and pathways between TM helix pairs 3 to 7 and 6 to 9 (37) have been reported to connect the wild-type protein to the cytoplasm. The water pathway observed in our study is located between TM helix pair 4 and 6 and has so far not been reported in the literature, which supports its proposed disease origin.

Leak currents have been described previously in reports of *ATP1A1* mutations in nonexcitable adenoma cells (13, 24) and in a study of *ATP1A1* mutations diagnosed in two patients with epilepsy (16). Leak currents in adenoma cells with *ATP1A* mutations were found to be too small to have functional consequences (24). By contrast, the leak currents found for the W931R mutant are very large (Fig. 4). Leak currents will have serious consequences in neurons since depolarization of the membrane potential, observed in neurons expressing mutated α1, can trigger epileptic activity.

Arginine (R)-rich peptides can generate water-filled pores in lipid bilayers that are cation selective (40). The mode of action remains elusive and has been informally referred to as “arginine magic.” According to a recent study, this arginine effect can be influenced by the membrane charge (41). In voltage-gated ion channels, arginine-mediated water pores play a major role for the movement of charges that determine the voltage sensitivity (42, 43). Mutations of critical arginine residues in voltage-gated ion channels are associated with leak currents, which can give rise to epilepsy (44) and peripheral paralysis (45). There are few, if any, previous studies that have demonstrated the electrogenic effects of substituting a neutral amino acid to arginine in excitable cells. The energy cost of directly bringing a positively charged arginine into the membrane is high (46). Nature has developed different mechanisms to insert arginine into the membrane, such as interactions with aromatic amino acids (47) or insertion *via* snorkeling (17, 48), which is the case for voltage-gated ion channels. Na,K-ATPase with the W931R mutation lacks such evolved structural features and was only partially integrated into the plasma membrane.

The lipid composition of the membrane can also affect both insertion and function of integral membrane proteins (49). It has previously been shown that folding and stabilization of Na,K-ATPase in the membrane depend on binding of a 18:0 to 18:1 phosphatidylserine (PS) and cholesterol at a site comprising residues in or near α trans-membrane segments 8 and 9 (25, 26, 27). W931 makes close contact with a bound cholesterol and phospholipid at this location (31). Introduction of a positively charged arginine may also have disturbed these specific lipid–protein interactions by affecting the normal folding of the C-terminal trans-membrane segments and contributing to the formation of the cation leakage pathway.

Our patient had hypomagnesemia due to urinary magnesium loss. Hypomagnesemia was also previously observed in children with *ATP1A1* variants and epilepsy (16). Mg balance is regulated by Mg reabsorption in the renal distal convoluted tubule (DCT) (50), driven by the apical membrane potential, which powers Mg entry into the cells *via* TRPM6 channels and basolateral Na,K-ATPase activity, which creates the Na gradient that drives Mg efflux *via* the Na/Mg exchange transporter, SLC41A1 (50, 51). The hypomagnesemia can be explained by reduced Na,K-ATPase abundance or activity in DCT, as discussed in relation to other examples of Mg wasting caused by inactivation or destabilization of Na,K-ATPase (52). Note, however, that loss of Na,K-ATPase activity does not itself suffice to explain the main CNS phenotype, which depends on the gain of toxic function, *i.e.*, the leak current.

Na,K-ATPase is crucial for cardiac cell electrophysiology.Yet our patient did not have signs of cardiac distress, and cardiac symptoms have not been noted in previously reported cases with ATP1A1 mutation. In the postnatal heart, α1 and α2 isoforms are both involved in regulation of cardiac contractility (52). In mice, downregulation of one α1 allele results in upregulation of the α2 allele (53). A compensatory upregulation of α2 might explain why our patient did not have signs of cardiac distress and why cardiac symptoms were not noted in previously reported cases with ATP1A1 mutation. Most neurons also express two isoforms, α1 and α3. Since these isoforms have different sodium affinity and are complementary (8), upregulation of α3 would not compensate for loss of α1 function. Nor would it neutralize the dysfunction of the mutated α3.

The vast majority of nonexcitable cells express only one α subunit. Yet the symptoms of affected infant were mainly confined to the central nervous system. This suggests that, under resting conditions and with little variation in food intake, the single WT α1 allele is sufficient to maintain the transmembrane sodium gradient and sodium-supported transport of ions and nutrients in nonneuronal cells. Deletion of the mutant allele could have improved the epileptic seizures in our patient but the application of gene therapy in neurological disorders is still in an experimental phase (54). Only a fraction of the mutant Na,K-ATPase was inserted into the plasma membrane. Complete inhibition of its membrane insertion could also have had a therapeutic effect. The lipid composition of the membrane affects the function and insertion of integral proteins. Improved knowledge of the role of different fatty acids in the integration of proteins with an arginine mutation might open up new pathways to treat certain forms of epilepsy caused by gain-of-function mutations (46). The water influx pathway into the mutant Na,K-ATPase α subunit might be a novel target for therapeutic intervention and should be investigated in future docking studies.

## Experimental procedures

### Human subjects

The study was approved by the Ethics Committee of Karolinska Institutet (Stockholm, Sweden), and written informed consent was obtained from the parents of the patient according to the Declaration of Helsinki. Genomic DNA was extracted from blood samples of the patient and her healthy unrelated parents.

### WGS

Paired-end WGS using HiSeq X (Illumina) was performed with a PCR-free library using a TruSeq DNA PCR-Free library preparation kit at Clinical Genomics, SciLifeLab. The libraries were sequenced to an average read depth of 30×. Single nucleotide variants (SNVs) and indels were called using the HaplotypeCaller in GATK (v3.7) (55). Rare variants with a minor allele frequency of less than 1% in ExAC (v0.2) (19) or the Swedish variant frequency database (20) were considered for further analysis. Finally, CADD (18) was used to score the deleteriousness of SNVs, and variants were manually evaluated according to different inheritance models. The candidate variant determined by WGS was confirmed by Sanger sequencing. For further details, see Supporting information (Text S2; Table S1).

### Sample preparation and imaging of brain paraffin sections

Paraffin-embedded brain sections were dewaxed and antigen retrieval was performed as described previously (56). The following primary antibodies were used for immunolabeling: a chicken polyclonal anti-MAP2 antibody (ab5392; Abcam), a rabbit monoclonal anti-Na,K-ATPase α subunit antibody (ab76020, Abcam), and a mouse monoclonal anti-Na,K-ATPase α1 subunit antibody (a6F; DSHB). Confocal microscopy was performed with Zeiss LSM 780 and Leica TCS SP8 microscopes. For further details, see Supporting information (Text S3).

### Sample preparation and imaging for membrane expression and cell survival experiments

Primary hippocampal neurons were derived from E18 Sprague Dawley rat embryos as described previously (8). Ethical permission for use of rat primary culture was obtained from Stockholm Norra Försöksdjursetiska nämnd (Dr Nr 1822-2020). Neurons were transfected after 21 days *in vitro* with plasmids encoding WT-Na,K-ATPase α1-GFP or W931R-Na,K-ATPase α1-GFP (57). For the survival studies, HEK 293a cells were transfected with plasmids encoding: ouabain-sensitive (OS), ouabain-resistant (OR), and ouabain resistant with variant (W931R-OR) Na,K-ATPase α1. After 24 h of transfection, cells were treated with ouabain 10 μM to inhibit the endogenous Na,K-ATPase activity. The number of cells was counted and normalized to untreated control.

Live-cell confocal images were acquired at 1, 2, and 3 days after transfection. Membrane expression of the respective protein was calculated by measuring the average fluorescence intensity in a cell-membrane-containing area and comparing it with the average fluorescence intensity in an area of the same size in the cytoplasm following subtraction of the background signal from both values. For nanobody labeling, 21-day-old rat hippocampal neurons were transfected with WT-Na,K-ATPase α1-GFP or W931R-Na,K-ATPase α1-GFP. After 3 days, live cells were stained for 5 min with an anti-GFP nanobody (GFP-Booster_Atto594, gba594-100; Chromotek) (Fig. S7). Confocal microscopy was performed with a Zeiss LSM 780 microscope. For further details, see Supporting information (Text S4).

### Membrane resting potential

For patch-clamp recordings, cells were recorded 2 to 5 days after transfection. Coverslips were placed in the recording chamber containing the extracellular solution (in mM: 110 NaCl, 1 NaH2PO4, 4 KCl, 25 HEPES, 10 Glucose, 1.2 MgCl2 and 1.2 CaCl2, pH 7,4 with NaOH) for a maximum of 1 h. Whole-cell current clamp recordings of cells were made with a patch electrode filled with a solution containing (in mM): 120 K-gluconate, 24 KCl, 4 NaCl, 4 MgCl2, 0.16 EGTA, 10 HEPES, and 4 K2-ATP, pH 7,2 with KOH, and the resting membrane potential was recorded using a Multiclamp 700B amplifier (Molecular Devices). Data were acquired using Clampex 10 (Molecular devices).

### Expression and activity analysis in *P. pastoris*

*Pichia pastoris* (strain SMD 1165) transformation, clone selection, yeast growth, induction of protein expression by methanol, and membrane preparation of wild-type human α1β1 and the mutant α1W931Rβ1 Na,K-ATPase were done essentially as described previously (58, 59). Variants were introduced into PhilD2 vector harboring human α_1_ and His10-β_1_ by overlap extension PCR (27, 60, 61). For Western blot analysis, 50 μg of membrane protein was separated by SDS-PAGE, transferred onto a nitrocellulose membrane, and detected using the anti-KETTY antibody, as described previously (58, 59).

Na,K-ATPase activity of yeast membranes was measured, after unmasking with 0.3 mg/ml SDS, using a PiColor Lock malachite green agent (Inova Biosciences) to detect free Pi, in a medium containing 130 mM NaCl, 20 mM KCl, 2 mM MgCl2, 0.5 mM EGTA, 25 mM histidine, pH 7.4, 1 mM Na-azide, and 0.8 mM ATP at 37 °C, without or with 200 μM ouabain (59, 61). For WT the ouabain-sensitive fraction of Pi release was about 70%. Ouabain binding to yeast membranes using [^3^H-ouabain] was done as described previously (62).

### Modeling and simulations

Models of human Na,K-ATPase including the α1, β1, and γ subunits were generated using MODELLER (63) based on the X-ray structure of the homologous pig renal complex in the Na^+^-bound state (PDB ID: 4HQJ) and K^+^-bound state (PDB ID: 3KDP). Residue numbering from the template structure is used throughout this section. The pathological variant (W924R in crystallographic numbering) was introduced with VMD software (64).

WT and mutant human Na,K-ATPase models were inserted into DOPC lipid bilayers and solvated using the TIP3P water model (65) and 0.15 mM NaCl with CHARMM-GUI (66). Each simulation system was energy-minimized for 10,000 steps, followed by heating to 310 K during a 500-ps NVT simulation, and finally a 1-ns NPT simulation with only the protein backbone restrained. After achieving a tight seal between protein and lipids, water molecules in the membrane–protein interface were removed. The protein was then energy-minimized again for 10,000 steps, followed by NPT production runs. Simulations were run with NAMD 2.10 (67) and CHARMM36 (68, 69) force fields.

The Na^+^ and K^+^-binding homology models were also embedded into a multicomponent, asymmetric lipid bilayer consisting of phospholipids (POPC, POPE, POPS, POPI), sphingolipid (SSM), glycolipids (GM3), and cholesterol (CHL), thereby mimicking a native plasma membrane (70, 71). The outer leaflet contained POPC:POPE:SSM:GM3:CHL in the ratio of 40:10:15:10:25 while the inner leaflet had POPC:POPE:POPS:POPI:CHL in the ratio of 10:40:15:10:25. We performed four parallel, independent production simulations, two each for the mutated E1-and E2-states for 500 ns followed by 100 ns with an applied 100 mV electrical field. One E1 simulation was extended further for another 400 ns (total 1000 ns). The plasma membrane simulations were run with the GROMACS-2019 package (72) and CHARMM36 force fields (68, 69).

### Two-electrode voltage-clamp electrophysiology

Constructs encoding the *ATP1A1* (human WT or W931R) and *ATP1B1* gene products were synthesized and subcloned into the pUNIV vector (73) and transcribed using a mMESSAGE mMACHINE T7 kit (Thermo Fisher Scientific). Isolated oocytes extracted from female *X. laevis* frogs were injected with 7 ng of *ATP1A1* mRNA and 1 ng of *ATP1B1* mRNA. Injected oocytes were stored individually at 12 °C for 4 to 6 days.

Two-electrode voltage-clamp recordings of Na,K-ATPase currents were performed under Vmax conditions according to previous protocols (74). Recordings were performed at −70 mV. Currents were digitized at a sampling rate of 5 kHz. Changes in the baseline current (in the absence of K^+^) were measured immediately upon voltage clamp and after 15 min. Results were analyzed by an ordinary one-way analysis of variance, with significance set to *p* < 0.05, using Prism 7 for Mac (GraphPad Software). For further details, see Supporting information (Text S5).

## Data availability

All data for this study are included within this article.

## Supporting information

This article contains supporting information (55, 57, 59, 61, 75, 76).

## Conflict of interest

The authors declare that they have no conflict of interest with the content of this article.

## References

[1] Galanopoulou A.S. (2013). Basic mechanisms of catastrophic epilepsy -- overview from animal models. Brain Dev..

[2] Barry J.M., Holmes G.L. (2016). Why are children with epileptic encephalopathies encephalopathic?. J. Child Neurol..

[3] Helbig I., Tayoun A.A.N. (2016). Understanding genotypes and phenotypes in epileptic encephalopathies. Mol. Syndromol..

[4] Richards K.L., Milligan C.J., Richardson R.J., Jancovski N., Grunnet M., Jacobson L.H., Undheim E.A.B., Mobli M., Chow C.Y., Herzig V., Csoti A., Panyi G., Reid C.A., King G.F., Petrou S. (2018). Selective NaV1.1 activation rescues Dravet syndrome mice from seizures and premature death. Proc. Natl. Acad. Sci. U. S. A..

[5] McTague A., Howell K.B., Cross J.H., Kurian M.A., Scheffer I.E. (2016). The genetic landscape of the epileptic encephalopathies of infancy and childhood. Lancet Neurol..

[6] Krishnan G.P., Filatov G., Shilnikov A., Bazhenov M. (2015). Electrogenic properties of the Na^+^/K^+^ ATPase control transitions between normal and pathological brain states. J. Neurophysiol..

[7] Attwell D., Laughlin S.B. (2001). An energy budget for signaling in the grey matter of the brain. J. Cereb. Blood Flow Metab..

[8] Azarias G., Kruusmägi M., Connor S., Akkuratov E.E., Liu X.-L., Lyons D., Brismar H., Broberger C., Aperia A. (2013). A specific and essential role for Na,K-ATPase α3 in neurons co-expressing α1 and α3. J. Biol. Chem..

[9] Sweadner K.J., Arystarkhova E., Penniston J.T., Swoboda K.J., Brashear A., Ozelius L.J. (2019). Genotype-structure-phenotype relationships diverge in paralogs ATP1A1, ATP1A2, and ATP1A3. Neurol. Genet..

[10] Heinzen E.L., Swoboda K.J., Hitomi Y., Gurrieri F., Nicole S., de Vries B., Tiziano F.D., Fontaine B., Walley N.M., Heavin S., Panagiotakaki E., Fiori S., European Alternating Hemiplegia of Childhood (AHC) Genetics Consortium, Biobanca e Registro Clinico per l'Emiplegia Alternante (I.B.AHC) Consortium, European Network for Research on Alternating Hemiplegia (ENRAH) for Small and Medium-sized Enterpriese (SMEs) Consortium (2012). *De novo* mutations in ATP1A3 cause alternating hemiplegia of childhood. Nat. Genet..

[11] Rosewich H., Ohlenbusch A., Maschke U., Altmüller J., Frommolt P., Zirn B., Ebinger F., Siemes H., Nürnberg P., Brockmann K., Gärtner J. (2012). Heterozygous de-novo mutations in ATP1A3 in patients with alternating hemiplegia of childhood: A whole-exome sequencing gene-identification study. Lancet Neurol..

[12] de Carvalho Aguiar P., Sweadner K.J., Penniston J.T., Zaremba J., Liu L., Caton M., Linazasoro G., Borg M., Tijssen M.A., Bressman S.B., Dobyns W.B., Brashear A., Ozelius L.J. (2004). Mutations in the Na+/K+ -ATPase alpha3 gene ATP1A3 are associated with rapid-onset dystonia parkinsonism. Neuron.

[13] Azizan E.A., Poulsen H., Tuluc P., Zhou J., Clausen M.V., Lieb A., Maniero C., Garg S., Bochukova E.G., Zhao W., Shaikh L.H., Brighton C.A., Teo A.E., Davenport A.P., Dekkers T. (2013). Somatic mutations in ATP1A1 and CACNA1D underlie a common subtype of adrenal hypertension. Nat. Genet..

[14] Beuschlein F., Boulkroun S., Osswald A., Wieland T., Nielsen H.N., Lichtenauer U.D., Penton D., Schack V.R., Amar L., Fischer E., Walther A., Tauber P., Schwarzmayr T., Diener S., Graf E. (2013). Somatic mutations in ATP1A1 and ATP2B3 lead to aldosterone-producing adenomas and secondary hypertension. Nat. Genet..

[15] Lassuthova P., Rebelo A.P., Ravenscroft G., Lamont P.J., Davis M.R., Manganelli F., Feely S.M., Bacon C., Šafka Brožková D., Haberlova J., Mazanec R., Tao F., C. Saghira, Abreu L., Courel S. (2018). Mutations in ATP1A1 cause dominant charcot-marie-tooth type 2. Am. J. Hum. Genet..

[16] Schlingmann K.P., Bandulik S., Mammen C., Tarailo-Graovac M., Holm R., Baumann M., König J., Lee J.J.Y., Drögemöller B., Imminger K., Beck B.B., Altmüller J., Thiele H., Waldegger S., Van’t Hoff W. (2018). Germline *de novo* mutations in ATP1A1 cause renal hypomagnesemia, refractory seizures, and intellectual disability. Am. J. Hum. Genet..

[17] Schow E.V., Freites J.A., Cheng P., Bernsel A., von Heijne G., White S.H., Tobias D.J. (2011). Arginine in membranes: The connection between molecular dynamics simulations and translocon-mediated insertion experiments. J. Membr. Biol..

[18] Kircher M., Witten D.M., Jain P., O'Roak B.J., Cooper G.M., Shendure J. (2014). A general framework for estimating the relative pathogenicity of human genetic variants. Nat. Genet..

[19] Lek M., Karczewski K.J., Minikel E.V., Samocha K.E., Banks E., Fennell T., O'Donnell-Luria A.H., Ware J.S., Hill A.J., Cummings B.B., Tukiainen T., Birnbaum D.P., Kosmicki J.A., Duncan L.E., Estrada K. (2016). Analysis of protein-coding genetic variation in 60,706 humans. Nature.

[20] Ameur A., Dahlberg J., Olason P., Vezzi F., Karlsson R., Martin M., Viklund J., Kähäri A.K., Lundin P., Che H., Thutkawkorapin J., Eisfeldt J., Lampa S., Dahlberg M., Hagberg J. (2017). SweGen: A whole-genome data resource of genetic variability in a cross-section of the Swedish population. Eur. J. Hum. Genet..

[21] Adzhubei I.A., Schmidt S., Peshkin L., Ramensky V.E., Gerasimova A., Bork P., Kondrashov A.S., Sunyaev S.R. (2010). A method and server for predicting damaging missense mutations. Nat. Methods.

[22] Ng P.C., Henikoff S. (2003). Sift: Predicting amino acid changes that affect protein function. Nucleic Acids Res..

[23] Jagadeesh K.A., Wenger A.M., Berger M.J., Guturu H., Stenson P.D., Cooper D.N., Bernstein J.A., Bejerano G. (2016). M-CAP eliminates a majority of variants of uncertain significance in clinical exomes at high sensitivity. Nat. Genet..

[24] Meyer D.J., Gatto C., Artigas P. (2019). Na/K pump mutations associated with primary hyperaldosteronism cause loss of function. Biochemistry.

[25] Cornelius F., Habeck M., Kanai R., Toyoshima C., Karlish S.J.D. (2015). General and specific lipid-protein interactions in Na,K-ATPase. Biochim. Biophys. Acta.

[26] Habeck M., Haviv H., Katz A., Kapri-Pardes E., Ayciriex S., Shevchenko A., Ogawa H., Toyoshima C., Karlish S.J.D. (2015). Stimulation, inhibition, or stabilization of Na,K-ATPase caused by specific lipid interactions at distinct sites. J. Biol. Chem..

[27] Habeck M., Kapri-Pardes E., Sharon M., Karlish S.J. (2017). Specific phospholipid binding to Na,K-ATPase at two distinct sites. Proc. Natl. Acad. Sci. U. S. A..

[28] Toustrup-Jensen M.S., Holm R., Einholm A.P., Schack V.R., Morth J.P., Nissen P., Andersen J.P., Vilsen B. (2009). The C terminus of Na+,K+-ATPase controls Na+ affinity on both sides of the membrane through Arg935. J. Biol. Chem..

[29] Nyblom M., Poulsen H., Gourdon P., Reinhard L., Andersson M., Lindahl E., Fedosova N., Nissen P. (2013). Crystal structure of Na+, K(+)-ATPase in the Na(+)-bound state. Science.

[30] Catterall W.A. (2017). Forty years of sodium channels: Structure, function, pharmacology, and epilepsy. Neurochem. Res..

[31] Kanai R., Ogawa H., Vilsen B., Cornelius F., Toyoshima C. (2013). Crystal structure of a Na + -bound Na + ,K + -ATPase preceding the E1P state. Nature.

[32] Takeuchi A., Reyes N., Artigas P., Gadsby D.C. (2008). The ion pathway through the opened Na+,K+-ATPase pump. Nature.

[33] Gadsby D.C., Bezanilla F., Rakowski R.F., De Weer P., Holmgren M. (2012). The dynamic relationships between the three events that release individual Na^+^ ions from the Na^+^/K^+^-ATPase. Nat. Commun..

[34] Gadsby D.C., Takeuchi A., Artigas P., Reyes N. (2009). Peering into an ATPase ion pump with single-channel recordings. Philos. Trans. R. Soc. Lond. B Biol. Sci..

[35] Kopec W., Loubet B., Poulsen H., Kandelia H. (2014). Molecular mechanism of Na(+),K(+)-ATPase malfunction in mutations characteristic of adrenal hypertension. Biochemistry.

[36] Takeuchi A., Reyes N., Artigas P., Gadsby D.C. (2009). Visualizing the mapped ion pathway through the Na,K-ATPase pump. Channels (Austin)..

[37] Čechová P., Berka K., Kubala M. (2016). Ion pathways in the Na+/K+-ATPase. J. Chem. Inf. Model..

[38] Poulsen H., Khandelia H., Morth J.P., Bublitz M., Mouritsen O.G., Egebjerg J., Nissen P. (2010). Neurological disease mutations compromise a C-terminal ion pathway in the Na+/K+-ATPase. Nature.

[39] Paulsen P.A., Jurkowski W., Apostolov R., Lindahl E., Nissen P., Poulsen H. (2013). The C-terminal cavity of the Na,K-ATPase analyzed by docking and electrophysiology. Mol. Membr. Biol..

[40] Armstrong C.T., Mason P.E., Anderson J.L.R., Dempsey C.E. (2016). Arginine side chain interactions and the role of arginine as a gating charge carrier in voltage sensitive ion channels. Sci. Rep..

[41] Verbeek S.F., Awasthi N., Teiwes N.K., Mey I., Hub J.S., Janshoff A. (2021). How arginine derivatives alter the stability of lipid membranes: Dissecting the roles of side chains, backbone and termini. Eur. Biophys. J..

[42] Moreau A., Gosselin-Badaroudine P., Chahine M. (2014). Molecular biology and biophysical properties of ion channel gating pores. Q. Rev. Biophys..

[43] Payandeh J., Scheuer T., Zheng N., Catterall W.A. (2011). The crystal structure of a voltage-gated sodium channel. Nature.

[44] Sokolov S., Scheuer T., Catterall W.A. (2005). Ion permeation through a voltage- sensitive gating pore in brain sodium channels having voltage sensor mutations. Neuron.

[45] Jiang D., Gamal El-Din T.M., Ing C., Lu P., Pomès R., Zheng N., Catterall W.A. (2018). Structural basis for gating pore current in periodic paralysis. Nature.

[46] Honig B.H., Hubbell W.L. (1984). Stability of “salt bridges” in membrane proteins. Proc. Natl. Acad. Sci. U. S. A..

[47] Hohlweg W., Wagner G.E., Hofbauer H.F., Sarkleti F., Setz M., Gubensäk N., Lichtenegger S., Falsone S.F., Wolinski H., Kosol S., Oostenbrink C., Kohlwein S.D., Zangger K. (2018). A cation–π interaction in a transmembrane helix of vacuolar ATPase retains the proton-transporting arginine in a hydrophobic environment. J. Biol. Chem..

[48] Ulmschneider J.P., Andersson M., Ulmschneider M.B. (2011). Determining peptide partitioning properties via computer simulation. J. Membr. Biol..

[49] Elinder F., Liin S.I. (2017). Actions and mechanisms of polyunsaturated fatty acids on voltage-gated ion channels. Front. Physiol..

[50] Schäffers O.J.M., Hoenderop J.G.J., Bindels R.J.M., de Baaij J.H.F. (2018). The rise and fall of novel renal magnesium transporters. Am. J. Physiol. Renal Physiol..

[51] Mayan H., Farfel Z., Karlish S.J.D. (2018). Renal Mg handling, FXYD2 and the central role of the Na,K-ATPase. Physiol. Rep..

[52] McDonough A.A., Velotta J.B., Schwinger R.H.G., Philipson K.D., Farley R.A. (2002). The cardiac sodium pump: Structure and function. Basic Res. Cardiol..

[53] Dostanic I., Schultz J.E.J., Lorenz J.N., Lingrel J.B. (2004). The α1 isoform of Na,K-ATPase regulates cardiac contractility and functionally interacts and co-localizes with the Na/Ca exchanger in heart. J. Biol. Chem..

[54] Ingusci S., Cattaneo S., Verlengia G., Zucchini S., Simonato M. (2019). A matter of genes: The hurdles of gene therapy for epilepsy. Epilepsy Curr..

[55] McKenna A., Hanna M., Banks E., Sivachenko A., Cibulskis K., Kernytsky A., Garimella K., Altshuler D., Gabriel S., Daly M., DePristo M.A. (2010). The genome analysis Toolkit: A MapReduce framework for analyzing next-generation DNA sequencing data. Genome Res..

[56] Shi S.R., Chaiwun B., Young L., Cote R.J., Taylor C.R. (1993). Antigen retrieval technique utilizing citrate buffer or urea solution for immunohistochemical demonstration of androgen receptor in formalin-fixed paraffin sections. J. Histochem. Cytochem..

[57] Liebmann T., Fritz N., Kruusmägi M., Westin L., Bernhem K., Bondar A., Aperia A., Brismar H. (2018). Regulation of neuronal Na,K-ATPase by extracellular scaffolding proteins. Int. J. Mol. Sci..

[58] Strugatsky D., Gottschalk K.-E., Goldshleger R., Bibi E., Karlish S.J.D. (2003). Expression of Na+,K+-ATPase in *Pichia pastoris*: Analysis of wild type and D369N mutant proteins by Fe2+-catalyzed oxidative cleavage and molecular modeling. J. Biol. Chem..

[59] Cohen E., Goldshleger R., Shainskaya A., Tal D.M., Ebel C., le Maire M., Karlish S.J.D. (2005). Purification of Na+,K+-ATPase expressed in *Pichia pastoris* reveals an essential role of phospholipid-protein interactions. J. Biol. Chem..

[60] Ho S.N., Hunt H.D., Horton R.M., Pullen J.K., Pease L.R. (1989). Site-directed mutagenesis by overlap extension using the polymerase chain reaction. Gene.

[61] Kapri-Pardes E., Haviv H., Mahmmoud Y., Ilan M., Khalfin-Penigel I., Carmeli S., Yarden O., Karlish S.J.D. (2011). Stabilization of the alpha2 isoform of Na,K-ATPase by mutations in a phospholipid binding pocket. J. Biol. Chem..

[62] Katz A., Lifshitz Y., Bab-Dinitz E., Kapri-Pardes E., Goldshleger R., Tal D.M., Karlish S.J. (2010). Selectivity of digitalis glycosides for isoforms of human Na,K-ATPase. J. Biol. Chem..

[63] Webb B., Sali A. (2014). Comparative protein structure modeling using MODELLER. Curr. Protoc. Bioinformatics.

[64] Humphrey W., Dalke A., Schulten K. (1996). Vmd: Visual molecular dynamics. J. Mol. Graph..

[65] Jorgensen W.L., Chandrasekhar J., Madura J.D., Impey R.W., Klein M.L. (1983). Comparison of simple potential functions for simulating liquid water. J. Chem. Phys..

[66] Jo S., Kim T., Iyer V.G., Im W. (2008). CHARMM-GUI: A web-based graphical user interface for CHARMM. J. Comput. Chem..

[67] Phillips J.C., Braun R., Wang W., Gumbart J., Tajkhorshid E., Villa E., Chipot C., Skeel R.D., Kalé L., Schulten K. (2005). Scalable molecular dynamics with NAMD. J. Comput. Chem..

[68] Klauda J.B., Venable R.M., Freites J.A., O'Connor J.W., Tobias D.J., Mondragon-Ramirez C., Vorobyov I., MacKerell A.D., Pastor R.W. (2010). Update of the CHARMM all-atom additive force field for lipids: Validation on six lipid types. J. Phys. Chem. B.

[69] Best R.B., Zhu X., Shim J., Lopes P.E.M., Mittal J., Feig M., Mackerell A.D. (2012). Optimization of the additive CHARMM all-atom protein force field targeting improved sampling of the backbone phi, psi and side-chain chi(1) and chi(2) dihedral angles. J. Chem. Theory Comput..

[70] Koldso H., Reddy T., Fowler P.W., Duncan A.L., Sansom M.S. (2016). Membrane compartmentalization reducing the mobility of lipids and proteins within a model plasma membrane. J. Phys. Chem. B.

[71] Harayama T., Riezman H. (2018). Understanding the diversity of membrane lipid composition. Nat. Rev. Mol. Cell Biol..

[72] Abraham M.J., Murtola T., Schulz R., Páll S., Smith J.C., Hess B., Lindahl E. (2015). Gromacs: High performance molecular simulations through multi-level parallelism from laptops to supercomputers. SoftwareX.

[73] Venkatachalan S.P., Bushman J.D., Mercado J.L., Sancar F., Christopherson K.R., Boileau A.J. (2007). Optimized expression vector for ion channel studies in Xenopus oocytes and mammalian cells using alfalfa mosaic virus. Pflugers Arch..

[74] Horisberger J.D., Kharoubi-Hess S. (2002). Functional differences between alpha subunit isoforms of the rat Na,K-ATPase expressed in Xenopus oocytes. J. Physiol..

[75] McLaren W. (2010). Deriving the consequences of genomic variants with the ensembl API and SNP effect predictor. Bioinformatics.

[76] Paila U., Chapman B.A., Kirchner R., Quinlan A.R. (2013). GEMINI: integrative exploration of genetic variation and genome annotations. PLoS Comput. Biol..

