# Supplementary material for: A missense mutation converts the Na+,K+-ATPase into an ion channel and causes therapy-resistant epilepsy
Source: J Biol Chem. 2021 Oct 28;297(6):101355. doi: 10.1016/j.jbc.2021.101355 (PMC8637647; doi:10.1016/j.jbc.2021.101355)
Supplement: Text S1–S5 and Figures S1–S7 Table S1 [file mmc1.pdf]

Supplementary Information

Text S1–S5

Figures S1–S7

Table S1

SI references

Text S1. Extensive metabolic investigations, including muscle biopsy, did not reveal any pathology. Newborn screening yielded normal results. Metabolic screening was performed by measuring plasma amino acids, the plasma acylcarnitine profile, plasma carnitine, plasma pipecolic acid, urine orotic acid, urine purines and pyrimidines, plasma carbohydrate-deficient transferrin, plasma creatine kinase, cerebrospinal fluid (CSF) amino acids, CSF lactate, and the CSF/plasma glucose ratio. All results were normal. The urinary level of total amino acids was increased, indicating tubular immaturity, which is a common finding in this age group. Urine organic acid profiles indicated an increased level of glutarate and a trace of 3-OH glutarate. Concomitantly, the homozygous mutation c.895C>T in *SUGCT* was found. The mutation is previously known and causes glutaric aciduria type III, a condition characterized by the abovementioned metabolic defect, but without a clinical phenotype (PMID: 18926513). A similar metabolic defect is seen in glutaric aciduria type I and therefore we sequenced all coding sequences, as well as exon/intron regions, of glutaryl-CoA dehydrogenase, but did not detect any mutations. In addition, glutaryl-CoA dehydrogenase enzymatic activity in leukocytes was normal. We concluded that the defect was purely biochemical and hence classified it as glutaric aciduria type III. Muscle biopsy was obtained from the anterior tibialis. ATP synthesis and individual complex activities were normal in isolated mitochondria. There were no mutations in mitochondrial DNA. Subsequent Southern blotting showed a normal restriction pattern.

During the long-lasting seizures the child was connected to a pulse, saturation, ECG monitor a great part of her life, and no cardiovascular incidents were reported. Her pulse was age appropriate, and the echocardiograms were normal. At autopsy her heart had normal dimensions, normal weight, and a visual inspection did not reveal any malformations.

Text S2. Genome analysis was performed using an in-house mutation identification pipeline (https://github.com/Clinical-Genomics/MIP) developed by Clinical Genomics to map reads to the human reference genome (GRCh37/hg19). SNVs and indels were called using the HaplotypeCaller in GATK (v3.7) (1). The variants were further processed and manipulated with GenotypeGVCFs, VariantRecalibrator, ApplyRecalibration, CalculateGenotypePosteriors, VariantFiltration and VariantAnnotator tools in GATK according to best practice guidelines, and then functionally annotated using Variant Effect Predictor (Ensembl 84) (2) and loaded into an SQL database using GEMINI (v0.19.0) (3). The variants were explored in the database using GEMINI built-in tools.

Text S3. Briefly, dewaxed sections were immersed in sodium citrate buffer (10 mM sodium citrate and 0.05% Tween 20, pH 6.0) heated to 100°C for 10 min. After cooling, the sections were incubated in PBS (137 mM NaCl, 3 mM KCl, 8 mM Na_2HPO_4, and 1.5 mM KH_2PO_4, pH 7.4) containing 0.5% Triton X-100 for 5 min. The sections were then blocked with PBS containing 5% bovine serum albumin and incubated overnight at 4°C with a primary chicken polyclonal anti-MAP2 antibody (ab5392; Abcam, Cambridge, UK) (dilution: 1:400), a primary rabbit monoclonal anti-Na,K-ATPase α subunit antibody (ab76020, Abcam) (dilution: 1:400), and a primary mouse monoclonal anti-Na,K-ATPase α1 subunit antibody (a6F; DSHB, Iowa City, IA, USA) (dilution: 1:400). After two washes with PBS for 1 h, the sections were incubated with goat anti-chicken IgY (H&L)-Alexa Fluor 488 (ab150173, Abcam) (dilution: 1:500), goat anti-rabbit IgG-Abberior STAR 635P (2-0012-007-2; Abberior, Göttingen, Germany) (dilution: 1:500), and goat anti-mouse IgG (H&L)-Alexa Fluor 594 (A-11032; Invitrogen, Carlsbad, CA, USA) (dilution: 1:500) secondary antibodies for 5 h. After immunolabeling, the samples were mounted in Mowiol supplemented with 0.1% (wt/vol) DABCO (1,4-diazabicyclo [2.2.2] octane; Sigma-Aldrich, St. Louis, MO, USA) and 2.5 µg/mL DAPI (4′,6-diamidino-2-phenylindole; Sigma-Aldrich). Confocal microscopy was performed with Zeiss LSM 780 and Leica TCS SP8 microscopes.

Text S4. Primary hippocampal neurons were transfected after 21 days *in vitro* with plasmids encoding WT-Na,K-ATPase α1-GFP and W931R-Na,K-ATPase α1-GFP (4), using Lipofectamine 2000 (11668019, Invitrogen). Live-cell confocal images were acquired at 1, 2, and 3 days after transfection. Membrane expression of the respective protein was calculated by

measuring the average fluorescence intensity in a cell membrane-containing area and comparing it with the average fluorescence intensity in an area of the same size in the cytoplasm following subtraction of the background signal from both values. For nanobody labeling, 21-day-old rat hippocampal neurons were transfected with WT-Na,K-ATPase α1-GFP or W931R-Na,K-ATPase α1-GFP. After 3 days, live cells were stained for 5 min with an anti-GFP nanobody (GFP-Booster_Atto594, gba594-100, Chromotek, Planegg-Martinsried, Germany) (dilution: 1:50). After washing with PBS, cells were fixed with prewarmed (37°C) PBS containing 4% (wt/vol) formaldehyde (P6148, Sigma-Aldrich) for 10 min. After washing with PBS, samples were mounted in Mowiol supplemented with 0.1% (wt/vol) DABCO (Sigma-Aldrich) and 2.5 μg/mL DAPI (Sigma-Aldrich). Confocal microscopy was performed with a Zeiss LSM 780 microscope.

Text S5. Briefly, oocytes were loaded with Na^+ by incubation overnight in loading medium (85 mM NaCl, 10 mM HEPES, 2.4 mM $NaHCO_3$, 0.4 mM $CaCl_2$, 0.8 mM $MgSO_4$, and 5 mM N-methyl-D-glucamine). Recordings were performed at -70 mV using an OC-725C voltage clamp (Warner Instruments, Hamden, CT, USA) in running buffer (100 mM sodium gluconate, 10 mM HEPES, 0.4 mM $CaCl_2$, 1 mM $MgCl_2$, 5 mM $BaCl_2$, and 10 mM tetraethylammonium chloride, adjusted to pH 7.5 with N-methyl-D-glucamine) at a flow rate of 0.5–1.0 mL/min. Activation buffers were serial dilutions of a 500 mM potassium gluconate stock solution generated using running buffer. Currents were digitized at a sampling rate of 5 kHz with an Axon CNS 1440A Digidata system using pCLAMP 10 (Molecular Devices, Sunnyvale, CA, USA). Changes in the baseline current (in the absence of K^+) were measured immediately upon voltage clamp and after 15 min. Results were analyzed by an ordinary one-way analysis of variance, with significance set to $P < 0.05$, using Prism 7 for Mac (GraphPad Software, La Jolla, CA, USA).

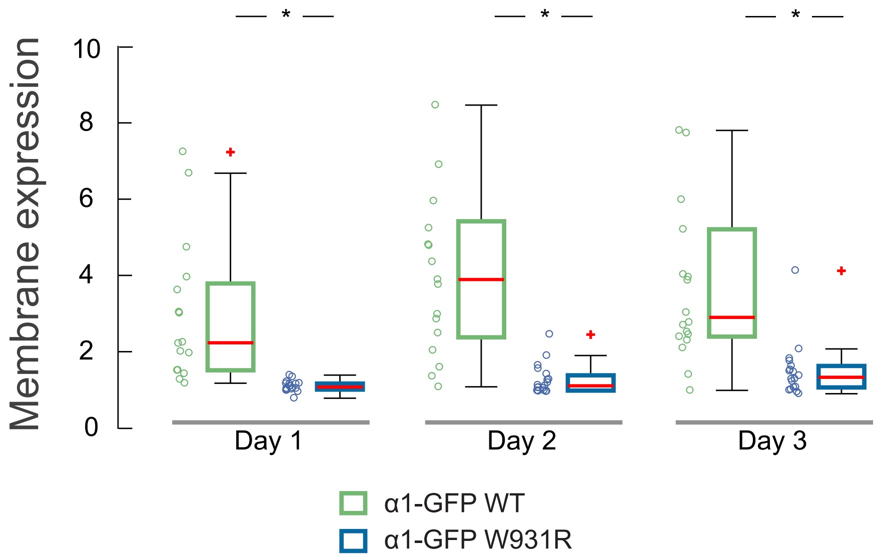

Figure S1. Quantification revealed that membrane expression of W931R α1 was significantly lower than that of WT α1 at 1, 2, and 3 days after transfection, showing that the mutation did not simply delay, but reduced, membrane expression. Wilcoxon rank sum test, $*p<0.001$.

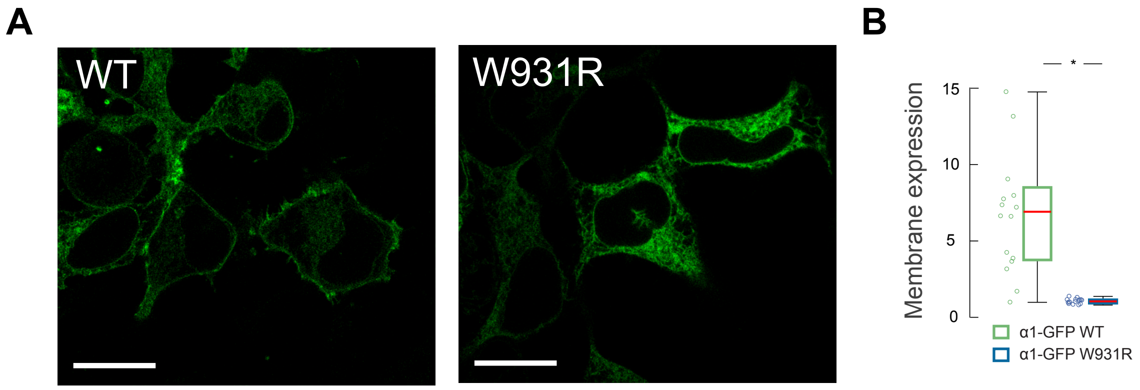

Figure S2. HEK293a cells expressing WT or W931R α1 genetically tagged with an extracellular GFP. (**A)** Representative images show that GFP localizes to the membrane in cells expressing WT α1, but this localization is less distinct in cells expressing W931R α1. Scale bars: 10 µm. (**B)** Membrane expression of the respective protein was calculated as the ratio of the average fluorescence intensity in the plasma membrane to the average fluorescence intensity in the cytoplasm corrected for background signals. Wilcoxon rank sum test, *$p<0.001$

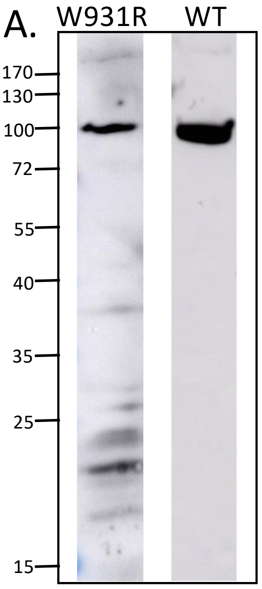

B.

	α1β1	α1W931Rβ1
**^{3}H-Ouabain binding pmole/mg**	18.0±1.0<br>N=4	0.31±0.1<br>N=4
**Na,K-ATPase activity µmoles/min/mg protein**	0.12±0.03<br>N=4	-0.015±0.008<br>N=4

Figure S3. Expression in Pichia pastoris, ouabain binding and Na,K-ATPase activity of α1W931Rβ1 compared with WT α1β1. (**A**) Western blots of expressed proteins using anti-KETTY. The blots display representative lanes from blots of α1W931Rβ1 expressed at 20°C compared to WT α1β1 expressed at 24°C for 48 hours. The blot illustrates expression of the α1W931R mutant at the optimal time and temperature, compared to the known optimal condition for expression of WT. At 24°C little or no full length α1W931R was detectable. Scans of several blots of WT and mutant show that the maximal amount of full length α1W931R, expressed at 20°C, was 20±2.9 % of WT (n=4). (**B**) ^{3}H-Ouabain binding and Na,K-ATPase activity in *P.pastoris* membranes. The mutant protein shows essentially no specific ouabain binding or Na,K-ATPase activity. WT activities are in the range described previously (6).

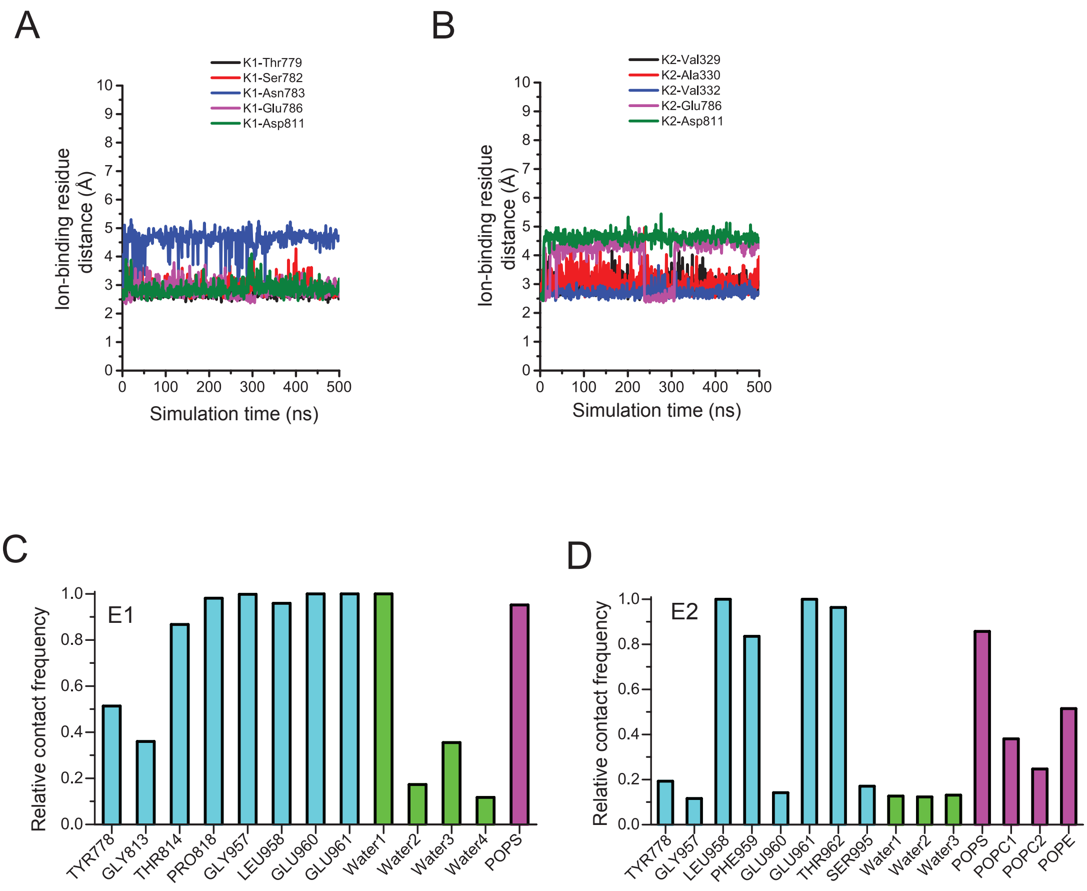

Figure S4. Simulated distances between K^+ ions and ion-coordinating residues in **(A)** site I and **(B)** site II in the mutant E2 state simulation. Chemical environment of the arginine mutation and ion-binding residues. Relative contact frequencies within 5 Å of the W931R mutation in the **(C)** E1 and **(D)** E2 states. Protein, water, and lipids are colored cyan, green, and magenta, respectively.

A

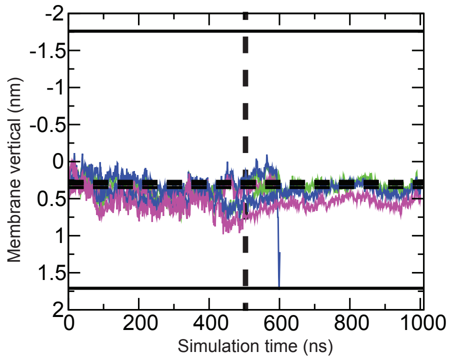

Figure S5. Vertical positions of Na^+ ions (E1 state). The average positions of the lipid phosphates are shown as solid lines. Horizontal dashed lines correspond to ion positions in the crystal structures. The vertical line indicates the onset of an applied 100 mV electric field.

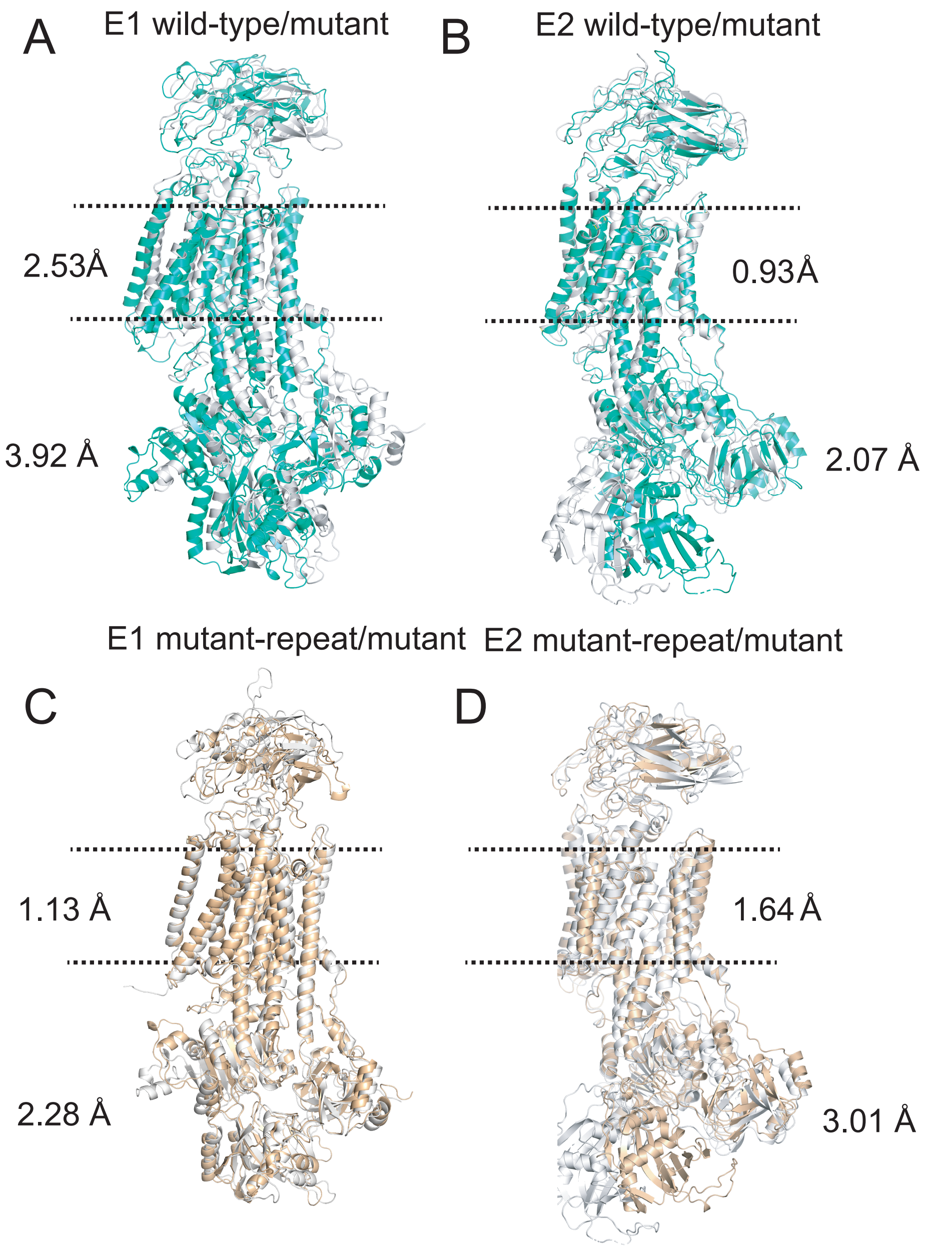

Figure S6. Superimposition of transmembrane (TM) sections of average structures from the final 50 ns of simulations. **A.** E1 WT (sea green) and mutant (gray). RMSD (TM) = 2.53 Å and RMSD (full protein) = 3.92 Å. **B.** E2 WT (sea green) and mutant (gray). RMSD (TM) = 0.93 Å and RMSD (full protein) = 2.07 Å. **C.** E1 mutant (gray) and mutant repeat simulation (wheat). RMSD (TM) = 1.13 Å and RMSD (full protein) = 2.28 Å. **D.** E2 mutant (gray) and mutant repeat simulation (wheat). RMSD (TM) = 1.64 Å and RMSD (full protein) = 3.01 Å.

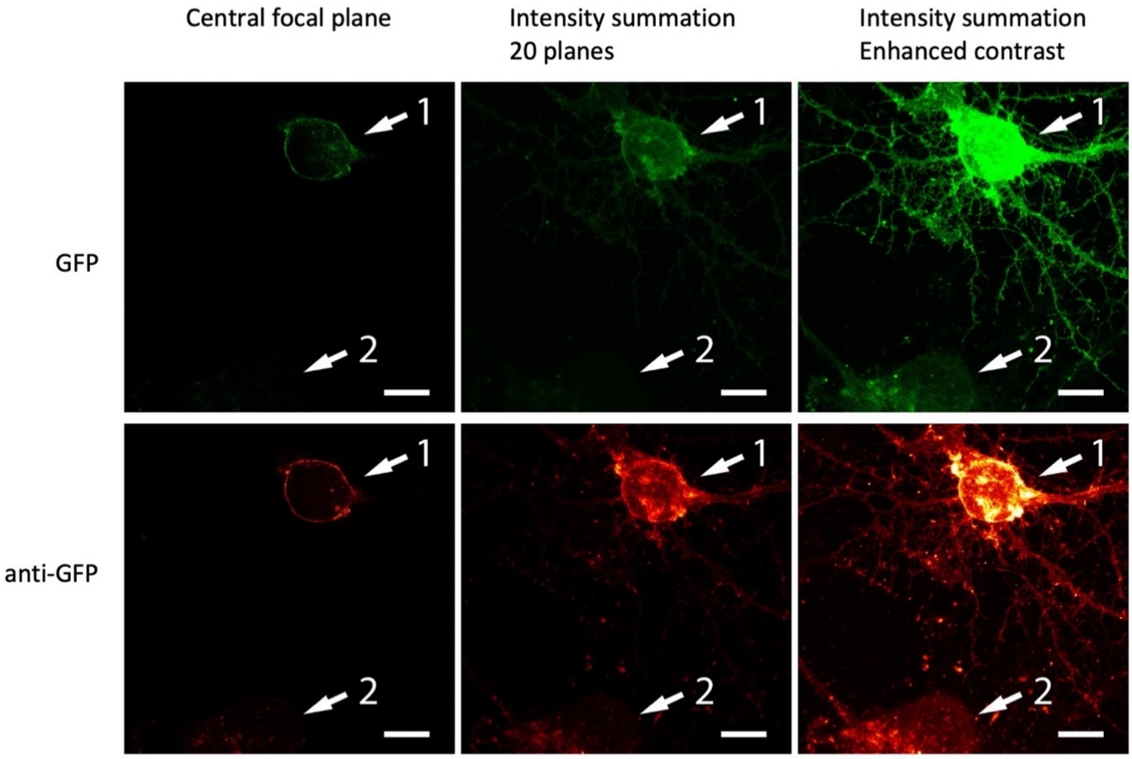

Figure S7. Confocal images of rat hippocampal neurons transfected with wild-type α1 tagged with GFP on the extracellular side. The images show a membrane localization of the GFP signal in a cell expressing wild-type α1-GFP (arrow 1). Labeling the live and unpermeabilized cells with a specific anti-GFP nanobody support the finding of a membrane localization of the protein. The anti-GFP nanobody labeling is specific since a cell which does not express wild-type α1-GFP (arrow 2, only observable by the weak autofluorescence in contrast enhanced images) exhibits very little unspecific anti-GFP nanobody labeling. Scale bar: 10 μm.

Chromosome	Start	End	Reference allele	Alternative allele	dbSNP identification number	ClinVar disease name	ClinVar variant identification numbe	GERP conservation score	GERP elements P-value	Gene	Transcript id and variant	ExAC minor allele frequency	ESP minor allele frequency	SweGen minor allele frequency	CADD scaled score	MCAP pathogenicity score	PolyPhen prediction	SIFT prediction	fitCons	Inheritance model
chr17	78059816	78059817	C	A	rs141343307	None	None	4,730000019	3,54E-34	*CCDC40*	NM_017950.3:c.2251C>A	0,002309	0,001585183	0,001	21	0,396578179	probably damaging	deleterious	0,615465	compound heterozygous / Autosomal recessive
chr17	78013850	78013851	A	G	rs201709592	Primary ciliary dyskinesia	RCV000152943.3|RCV000196597.1	-5,460000038	None	*CCDC40*	NM_019020.3:c.-4320T>C	0,002317	0,002708915	0,0045	8,12	0,00147867	benign	tolerated low confidence	0,706298	compound heterozygous / Autosomal recessive
chr7	42005387	42005388	C	T	rs370778213	None	None	5,46999979	2,33E-223	*GLI3*	NM_000168.5:c.3283G>A	3,30E-05	None	None	33	0,040893199	probably damaging	deleterious	0,719381	compound heterozygous / Autosomal recessive
chr7	42007505	42007506	G	A	rs121917716	Greig cephalopolysyndactyly syndrome	RCV000014843.25	4,940000057	5,25E-264	*GLI3*	NM_000168.5:c.2119C>T	0,0001977	0,000230663	None	24,9	0,157822136	probably damaging	deleterious	0,695654	compound heterozygous / Autosomal recessive
chr14	64688392	64688393	G	A	rs140897155	None	None	1,139999986	2,75E-07	*SYNE2*	NM_015180.4:c.20092G>A	0,001128	0,000999539	0,0055	6,89	0,00684711	benign	tolerated	0,706548	homozygous / Autosomal recessive
chr7	40498795	40498796	C	T	rs137852860	Glutaryl-CoA oxidase deficiency	RCV000001923.3	2,460000038	3,02E-08	*SUGCT*	NM_024728.2:c.895C>T	0,005359	0,005470085	0,016	13,85	0,128627862	probably damaging	tolerated	0,638212	homozygous / Autosomal recessive
chr1	116943823	116943824	T	C	None	None	None	5,099999905	5,24E-116	*ATP1A1*	NM_000701.7:c.2791T>C	None	None	None	23,9	0,590967129	probably damaging	deleterious	0,706548	De novo
chr17	16068339	16068340	C	CG	None	None	None	4,050000191	1,09E-152	*NCOR1*	NM_006311.2:c.571_572insC	None	None	None	None	None	None	None	0,732398	De novo

Table S1. Rare variants remaining after variant-filtering of whole-genome sequencing data.

SI references

1. A. McKenna *et al.*, The Genome Analysis Toolkit: a MapReduce framework for analyzing next-generation DNA sequencing data. *Genome Res* **20**, 1297-1303 (2010).
2. W. McLaren *et al.*, Deriving the consequences of genomic variants with the Ensembl API and SNP Effect Predictor. *Bioinformatics* **26**, 2069-2070 (2010).
3. U. Paila, B. A. Chapman, R. Kirchner, A. R. Quinlan, GEMINI: integrative exploration of genetic variation and genome annotations. *PLoS Comput Biol* **9**, e1003153 (2013).
4. T. Liebmann *et al.*, Regulation of Neuronal Na,K-ATPase by Extracellular Scaffolding Proteins. *Int J Mol Sci* **19** (2018).
5. Kapri-Pardes E, et al., Stabilization of the alpha2 isoform of Na,K-ATPase by mutations in a phospholipid binding pocket. J Biol Chem **286**(50):42888-42899 (2011).
6. Cohen E, et al., Purification of Na+,K+-ATPase expressed in Pichia pastoris reveals an essential role of phospholipid-protein interactions. J Biol Chem **280**(17):16610–16618 (2005).
